# ETV4 Promotes Colorectal Cancer Progression by Reprogramming Asparagine Metabolism to Remodel the Stromal Microenvironment

**DOI:** 10.1002/advs.202516557

**Published:** 2026-03-20

**Authors:** Dujiang Fu, Meijia Zhang, Maoping Cai, Miao Wang, Ziao Huang, Zesheng Lin, Yixin Liu, Wang Qian, Guopeng Chen, Yuxing Liang, Dongyi Wei, Jing xie, Picheng Yan, Yuanyuan Qu, Yongchang Wei

**Affiliations:** ^1^ Department of Radiation and Medical Oncology Hubei Cancer Clinical Study Center & Hubei Key Laboratory of Tumor Biological Behaviors Zhongnan Hospital of Wuhan University Wuhan China; ^2^ Department of Urology Fudan University Shanghai Cancer Center Shanghai China; ^3^ Department of Oncology Shanghai Medical College, Fudan University Shanghai China; ^4^ Department of Radiotherapy Yunnan Cancer Hospital, The Third Affiliated Hospital of Kunming Medical University Peking University Cancer Hospital Yunnan Kunming China; ^5^ Department of Urology Nanfang Hospital Southern Medical University Guangzhou China

**Keywords:** asparagine metabolism, colorectal cancer, ETV4, HGF/MET signaling, stromal microenvironment

## Abstract

Colorectal cancer (CRC) lethality is largely driven by liver metastasis and the associated tumor microenvironment (TME). This study identifies ETS variant transcription factor 4 (ETV4) as a central integrator of oncogenic signaling, metabolism, and stromal remodeling. In CRC cells, hepatocyte growth factor (HGF)/MET signaling induces ETV4 via an ERK1/2–p65 pathway. ETV4, in turn, directly activates MET and asparagine synthetase (ASNS), creating a positive feedback loop that amplifies MET signaling and elevates intracellular asparagine (Asn). Tumor‐derived Asn acts as a paracrine signal that induces inflammatory cancer‐associated fibroblast (iCAF) like activation in hepatic stellate cells (HSCs) and promotes iCAF polarization in primary CAFs, leading to enhanced HGF secretion that further stimulates MET^+^ tumor cells. Genetic and pharmacologic disruption of this axis attenuates CRC growth and metastatic traits in vitro and in mouse models. Notably, combined inhibition of HGF/MET signaling and Asn metabolism produces greater antitumor activity than either monotherapy. Together, these data delineate an HGF/MET → ETV4 → MET/ASNS → asparagine → iCAFs and iCAF‐like HSCs → HGF circuit that links signal amplification, metabolic reprogramming, and niche conditioning, and provide a rationale for therapeutic strategies co‑targeting HGF/MET and Asn pathways in advanced CRC.

## Graphical abstract

ETV4 coordinates a dual transcriptional program by upregulating MET and asparagine synthetase (ASNS). The resulting asparagine (Asn) secretion acts as a metabolic signal, reprogramming hepatic stellate cells (HSCs) and primary cancer‐associated fibroblasts (CAFs) toward an inflammatory phenotype. These activated stromal cells release HGF to sustain the MET–ERK1/2–NF‐κB–ETV4 axis, establishing a positive feedback loop that promotes CRC growth and liver metastasis.

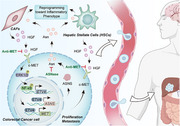

## 1 Introduction

Metastasis to distant organs, primarily the liver, is the principal cause of mortality in colorectal cancer (CRC) [1, 2]. Metastatic progression is not solely driven by tumor‐intrinsic properties but instead depends on dynamic interactions between cancer cells and the tumor microenvironment (TME) [3, 4]. Increasing evidence supports an ecosystem‐based model in which stromal components, including cancer‐associated fibroblasts (CAFs), influence metastatic colonization [4, 5]. In the liver, CAFs derived from hepatic stellate cells (HSCs) are associated with the establishment of a pro‐metastatic niche, in part through the secretion of growth factors and inflammatory mediators that can support tumor cell seeding and expansion [6, 7].

Two fundamental features of cancer—aberrant signal transduction and metabolic reprogramming—are central drivers of metastatic competence [8, 9]. As a well‐documented oncogenic driver, the hepatocyte growth factor (HGF)/MET receptor tyrosine kinase (RTK) axis promotes invasion and angiogenesis in many tumors [10, 11], and is of particular relevance in the HGF‐rich liver microenvironment [12]. However, the clinical efficacy of MET inhibitors as monotherapies is often hampered by intrinsic or acquired resistance, underscoring the need to understand the complex network regulating this pathway [13]. Concurrently, metabolic reprogramming, particularly of amino acids, has emerged as a crucial aspect of metastasis [14]. Among these, asparagine (Asn) bioavailability has been identified as a rate‐limiting factor for breast cancer metastasis, and its synthesizing enzyme, asparagine synthetase (ASNS), is upregulated in various tumors to support survival under nutrient stress [15, 16, 17]. Notably, whether tumor‐derived metabolites can act as extracellular signaling cues—beyond their cell‐intrinsic metabolic functions—to reprogram stromal cell states has not been systematically explored. Addressing this gap is critical for understanding how metastatic niches are dynamically established and maintained.

Transcription factors are ideal nodes for integrating upstream signals with downstream gene expression programs. ETV4 (ETS variant transcription factor 4), a known downstream effector of the RAS–MAPK pathway (a primary conduit for HGF/MET signaling), has been implicated in driving invasive programs in cancers like liver cancer [18], making it a promising candidate to bridge the aforementioned signaling and metabolic pathways. However, the functional role of ETV4 in CRC growth and metastasis, as well as its contribution to tumor–microenvironment crosstalk, remains poorly defined.

In this study, we identify ETV4 as a central regulator of CRC growth and liver metastasis. We demonstrate that ETV4 expression is driven by the HGF/MET–ERK1/2–p65 axis and, in turn, transcriptionally activates its upstream receptor MET, establishing a positive feedback loop. Furthermore, ETV4 directly activates the transcription of ASNS, thereby driving Asn biosynthesis. Importantly, we uncover an intercellular communication mechanism whereby tumor‐derived Asn acts as a paracrine metabolic signal that drives HSCs toward an inflammatory CAF (iCAF) like state and promotes iCAF polarization of CAFs, thereby enhancing HGF secretion and tumor progression. Based on this mechanism, we validate that combined targeting of HGF/MET signaling and Asn synergistically suppresses CRC growth and metastasis in preclinical models. Together, our study defines a novel ETV4‐centric regulatory network that integrates signal amplification, metabolic reprogramming, and microenvironment crosstalk, offering mechanistic insights and a clinically translatable therapeutic strategy for CRC.

## 2 Results

### 2.1 ETV4 is Frequently Upregulated in Colorectal Cancer and Correlates with Aggressive Features and Poor Prognosis

**FIGURE 1:**
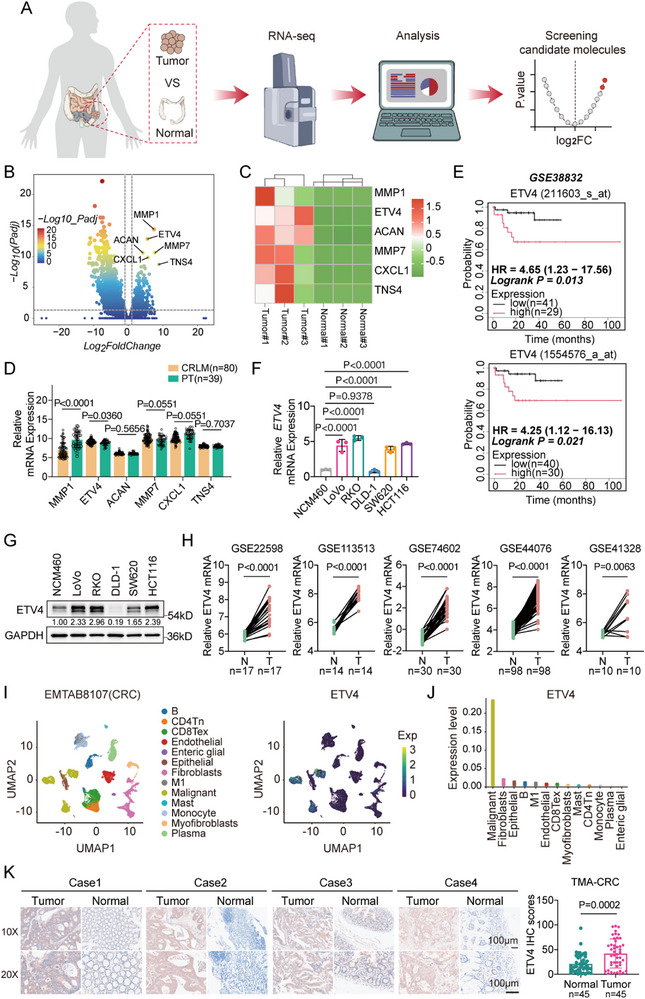
ETV4 is highly expressed in colorectal cancer (CRC) and associated with tumor progression and poor prognosis. (A) Schematic workflow of bulk RNA sequencing (RNA‐seq) in paired primary CRC tumors and adjacent normal tissues to identify candidate genes driving CRC progression. (B) Volcano plot showing differentially expressed genes (|log2Fold Change (FC)| > 1, adjusted *p* < 0.05), with the top six upregulated genes (MMP1, ETV4, ACAN, MMP7, CXCL1, and TNS4) highlighted. (C) Heatmap illustrating the expression of selected upregulated genes in three paired CRC tumor and normal samples. (D) Relative mRNA expression of the six candidate genes in primary CRC tumors (PT, *n* = 39) and CRC liver metastases (CRLM, *n* = 80) from the GSE41568 dataset. (E) Kaplan–Meier analysis of overall survival in CRC patients stratified by high versus low ETV4 expression (*n* indicated in the figure). (F,G) ETV4 expression in CRC cell lines: reverse transcription quantitative PCR (RT‐qPCR) analysis (*n* = 3) of mRNA levels relative to NCM460 (F) and Western blot of protein levels (G). (H) Relative ETV4 mRNA expression in paired normal (N) and CRC tumor (T) tissues across five independent GEO datasets (n indicated in the figure). (I,J) Single‐cell RNA‐seq (scRNA‐seq) analysis (EMTAB8107): Uniform Manifold Approximation and Projection (UMAP) visualization of cell clusters in the CRC tumor microenvironment (TME) (I, left), with ETV4 expression overlaid (I, right), and average ETV4 expression quantified across cell populations (J). (K) Representative immunohistochemistry (IHC) images and quantification of ETV4 staining in 45 paired CRC samples (TMA‐CRC cohort). Scale bar, 100 µm. Data are presented as mean ± SD (D,F,K). Statistical significance was determined by two‐tailed unpaired Student's *t*‐test (D), log‐rank test (E), one‐way ANOVA with Dunnett's multiple comparisons test (F), or two‐tailed paired Student's *t*‐test (H,K). *p* values are indicated in the figure, and *p* < 0.05 was considered statistically significant.

To identify genes associated with CRC progression, we performed RNA sequencing (RNA‐seq) on three paired primary CRC tumors and adjacent normal tissues (Figure 1A). Differential expression analysis revealed a set of significantly dysregulated genes, among which six were consistently upregulated across all tumor samples (Figure 1B,C). In addition, we assessed the expression levels of these six genes in an independent cohort of primary CRC tumors and liver metastases. Among these candidates, only ETV4 maintained significant overexpression in metastatic lesions compared to primary tumors (Figure 1D).

We validated this finding across multiple independent datasets. Analysis of Gene Expression Omnibus (GEO) datasets (GSE22598, GSE113513, GSE74602, GSE44076, and GSE41328) showed higher ETV4 mRNA levels in CRC tumors relative to paired normal tissues (Figure 1H). Similarly, data from The Cancer Genome Atlas (TCGA), including colon adenocarcinoma (COAD), rectal adenocarcinoma (READ), and combined CRC cohorts, demonstrated increased ETV4 expression in tumors (Figure S1A–C). In addition, ETV4 expression was higher in patients with distant metastasis (M1) compared to those without (M0) (Figure S1D). Kaplan–Meier survival analysis further showed that higher ETV4 expression was associated with shorter overall survival (Figure 1E).

At the cellular level, both reverse transcription quantitative PCR (RT‐qPCR) and Western blot analyses showed that ETV4 mRNA and protein levels were elevated across a panel of CRC cell lines compared to the nonmalignant colorectal epithelial cell line NCM460 (Figure 1F,G). Analysis of single‐cell RNA‐seq (scRNA‐seq) data (EMTAB8107) revealed that ETV4 expression was predominantly localized to malignant epithelial cells within the TME (Figure 1I,J). Immunohistochemistry (IHC) analysis of 45 paired CRC samples showed strong ETV4 staining in tumor cells, whereas adjacent normal tissues displayed minimal expression. Quantitative analysis of IHC scores from these samples showed higher ETV4 protein levels in tumors (Figure 1K). Therefore, these data indicate that ETV4 upregulation is associated with aggressive tumor features and poor clinical outcome in CRC (Table S6).

### 2.2 ETV4 Enhances CRC Growth and Metastatic Capacity In Vitro and In Vivo

**FIGURE 2:**
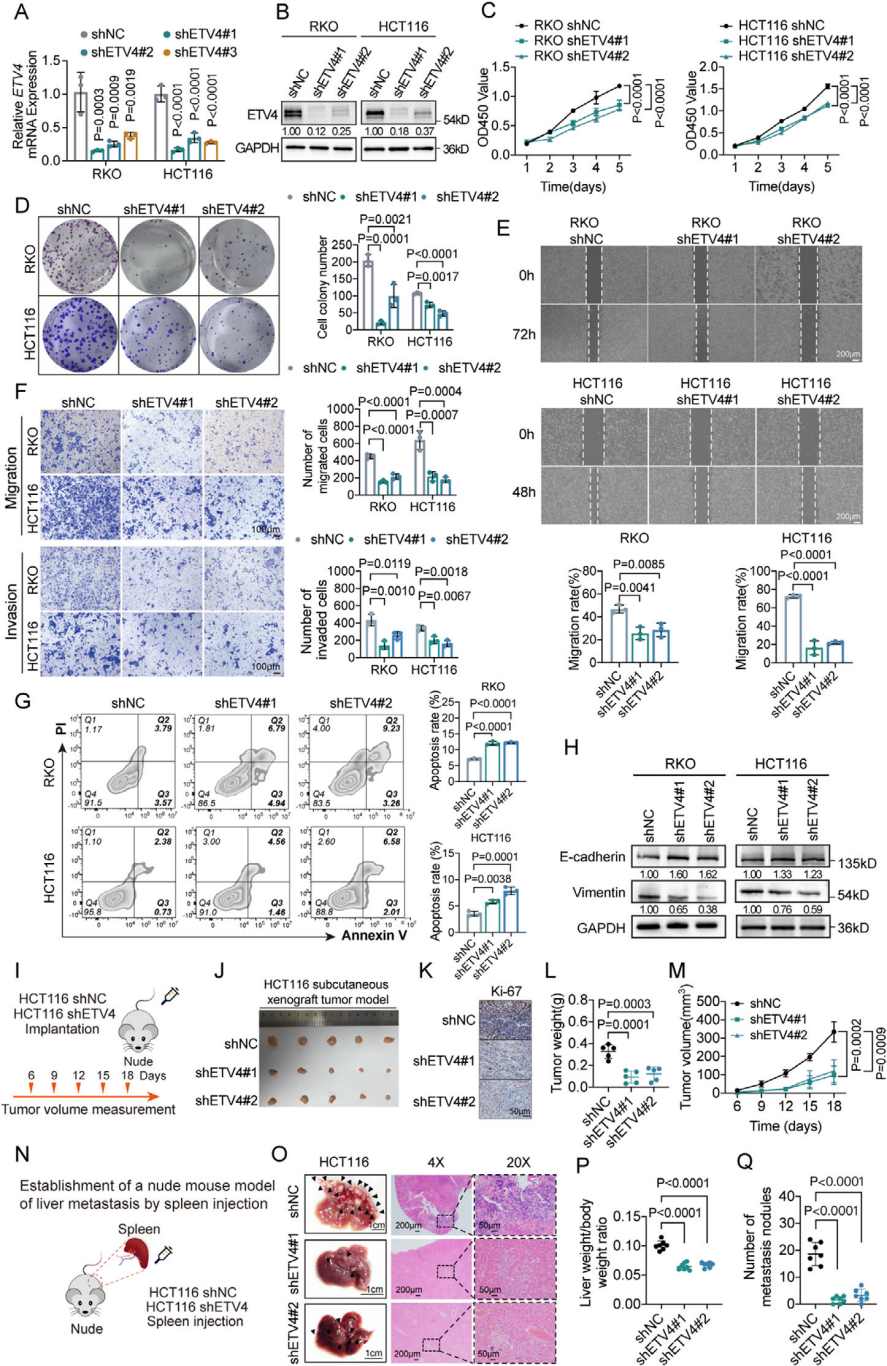
ETV4 silencing inhibits colorectal cancer (CRC) cell proliferation, migration, and invasion in vitro and suppresses tumor growth and liver metastasis in vivo. (A,B) Reverse transcription quantitative PCR (RT‐qPCR) (A, *n* = 3) and Western blot (B) analysis confirming ETV4 knockdown efficiency in RKO and HCT116 cells transfected with control shRNA (shNC) or ETV4‐targeting shRNAs (shETV4). (C–G) Functional analyses of shNC‐ and shETV4‐treated RKO and HCT116 cells, including CCK‐8 cell proliferation assays (C), colony formation assays (D), wound healing assays (E), Transwell migration and invasion assays (F), and Annexin V‐FITC/PI apoptosis assays (G). Quantification is provided for these panels (*n* = 3). Scale bars, 200 µm (E) and 100 µm (F). (H) Western blot analysis of the epithelial‐mesenchymal transition (EMT) markers E‐cadherin and Vimentin. (I) Schematic of the subcutaneous xenograft model design. (J) Images of xenograft tumors at the experimental endpoint. (K) Ki‐67 immunohistochemistry (IHC) staining of tumor sections. Scale bar, 50 µm. (L,M) Tumor weight (L) and tumor growth curves (M) (*n* = 5 mice per group). (N) Schematic of the intrasplenic injection liver metastasis model. (O) Representative gross images of livers (scale bar, 1 cm) and hematoxylin and eosin (H&E) stained sections at low (4×, scale bar, 200 µm) and high (20×, scale bar, 50 µm) magnifications. (P,Q) Quantification of liver‐to‐body weight ratio (P) and number of liver metastatic nodules (Q) (*n* = 7 mice per group). Data are presented as mean ± SD (A,C–G,L,M,P,Q). Statistical significance was determined by one‐way ANOVA with Dunnett's multiple comparisons test (A,D–G,L,P,Q), or two‐way ANOVA with Dunnett's multiple comparisons test (C,M). *p* values are provided in the figure, and *p* < 0.05 was considered statistically significant.

To investigate the functional role of ETV4, we established stable knockdown cell lines in RKO and HCT116 cells, which exhibit high endogenous ETV4, and stable overexpression lines in DLD‐1 cells, which have low endogenous ETV4 (Figure 2A,B; Figure S2A,B). In vitro, ETV4 knockdown significantly inhibited CRC cell proliferation, migration, and invasion, whereas its overexpression elicited the opposite effects (Figure 2C–F; Figure S2C–F). Furthermore, ETV4 silencing induced apoptosis and reversed the epithelial‐mesenchymal transition (EMT) phenotype, as evidenced by increased E‐cadherin and decreased Vimentin, and the opposite changes upon overexpression (Figure 2G,H; Figure S2G,H).

To validate these findings in vivo, we utilized xenograft models. Stable ETV4 knockdown in HCT116 cells markedly suppressed subcutaneous tumor growth, which was accompanied by a reduced Ki‐67 proliferation index (Figure 2I–M). In a liver metastasis model, HCT116 cells with ETV4 silencing formed significantly fewer and smaller metastatic nodules, resulting in a lower overall metastatic burden (Figure 2N–Q). Conversely, ETV4 overexpression in DLD‐1 cells led to accelerated tumor growth and an increased metastatic load in the liver (Figure S2I–O). Furthermore, in a syngeneic immunocompetent model (C57BL/6 mice), ETV4 silencing in MC38 cells significantly suppressed both subcutaneous tumor growth and liver metastasis formation (Figure S3A–G). These results confirm ETV4 as a potent driver of CRC growth and liver metastasis.

### 2.3 ASNS and MET are Direct Transcriptional Targets of ETV4 and Functionally Mediate its Oncogenic Effects

**FIGURE 3:**
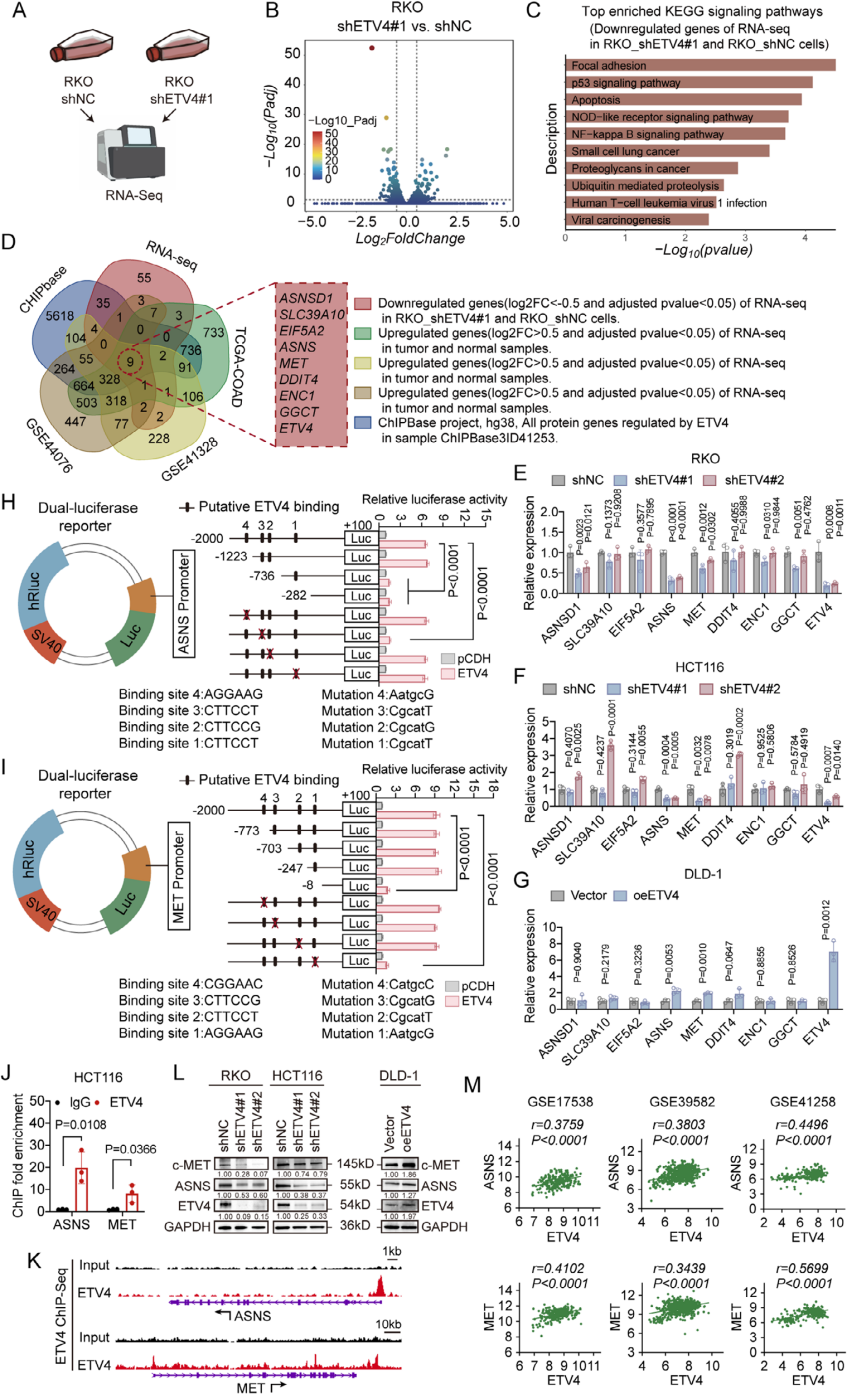
Asparagine synthetase (ASNS) and MET are direct transcriptional targets of ETV4 in colorectal cancer (CRC). (A) Schematic of the RNA sequencing (RNA‐seq) analysis comparing shETV4#1‐RKO and shNC‐RKO cells. (B) Volcano plot showing differentially expressed genes upon ETV4 knockdown (|log2Fold Change (FC)| > 0.5, adjusted *p* < 0.05). (C) KEGG pathway enrichment analysis of genes downregulated in shETV4#1‐RKO cells. (D) Venn diagram showing the overlap of genes downregulated in our RNA‐seq, upregulated in public CRC datasets, and predicted as ETV4 targets from ChIPBase. (E–G) Reverse transcription quantitative PCR (RT‐qPCR) analysis of selected candidate genes in ETV4‐silenced RKO and HCT116 cells (E,F) and ETV4‐overexpressing DLD‐1 cells (G) (*n* = 3). (H,I) Dual‐luciferase reporter assays in HCT116 cells cotransfected with an ETV4 expression vector and promoter constructs for ASNS (H) or MET (I) (*n* = 3). (J) ChIP‐qPCR showing ETV4 enrichment at the ASNS and MET promoter regions in HCT116 cells (*n* = 3). (K) ChIP‐seq tracks from an ENCODE dataset (accession: ENCSR714YZG) illustrating ETV4 binding peaks at the ASNS and MET promoters. (L) Western blot analysis of ASNS and MET protein levels in ETV4‐silenced and ETV4‐overexpressing CRC cells. (M) Correlation analysis of ASNS, MET, and ETV4 mRNA expression in public CRC datasets: GSE17538 (*n* = 232), GSE39582 (*n* = 585), and GSE41258 (*n* = 252). Pearson correlation coefficients (*r*) and *p* values are shown. Data are presented as mean ± SD (E–J). Statistical significance was determined by two‐tailed unpaired Student's *t*‐test (G,J), one‐way ANOVA with Dunnett's multiple comparisons test (E,F,H,I), or two‐sided Pearson correlation (M). *p* values are indicated in the figure, and *p* < 0.05 was considered statistically significant.

To elucidate the molecular basis underlying the pro‐tumorigenic role of ETV4, we performed RNA‐seq on ETV4‐silenced RKO cells. Gene set enrichment analysis (GSEA) revealed that genes downregulated upon ETV4 knockdown were significantly enriched in pathways associated with malignant phenotypes, including NF‐κB signaling, apoptosis, and focal adhesion (Figure 3A–C).

To identify candidate transcriptional targets of ETV4, we integrated our RNA‐seq data with public CRC tumor datasets and predicted ETV4 targets from ChIPBase, which identified eight candidate genes (Figure 3D). Among these, ASNS and the RTK MET showed consistent changes at both the mRNA and protein levels upon ETV4 knockdown or overexpression (Figure 3E–G,L). We therefore prioritized ASNS and MET for further validation. Bioinformatic analysis identified four putative ETV4 binding motifs within the promoter regions of ASNS and MET (Figures S9 and S10). Dual‐luciferase reporter assays demonstrated that ETV4 overexpression significantly increased the transcriptional activity of both promoters, whereas deletion of specific promoter regions (−1223 to −736 bp for ASNS and −247 to −8 bp for MET) markedly attenuated this effect. Furthermore, site‐directed mutagenesis identified specific ETV4 binding motifs—site 3 in the ASNS promoter and site 1 in the MET promoter—as essential for this transcriptional activation (Figure 3H,I). This direct binding was further validated by chromatin immunoprecipitation (ChIP)‐qPCR and analysis of ChIP‐seq data, which demonstrated ETV4 occupancy at conserved motifs within the ASNS and MET promoters (Figure 3J,K).

Functionally, silencing either ASNS or MET phenocopied the effects of ETV4 knockdown, resulting in reduced cell viability and clonogenicity, impaired migratory and invasive capacity, and increased apoptosis (Figure S4A–K). Moreover, expression of ASNS and MET positively correlated with ETV4 levels across multiple CRC patient datasets (Figure 3M). Collectively, these results support ASNS and MET as transcriptional targets of ETV4 and suggest that they contribute to ETV4‐associated oncogenic phenotypes in CRC.

### 2.4 ETV4 Promotes CRC Cell Proliferation and Migration by Transcriptionally Upregulating ASNS and Altering Asparagine Metabolism

**FIGURE 4:**
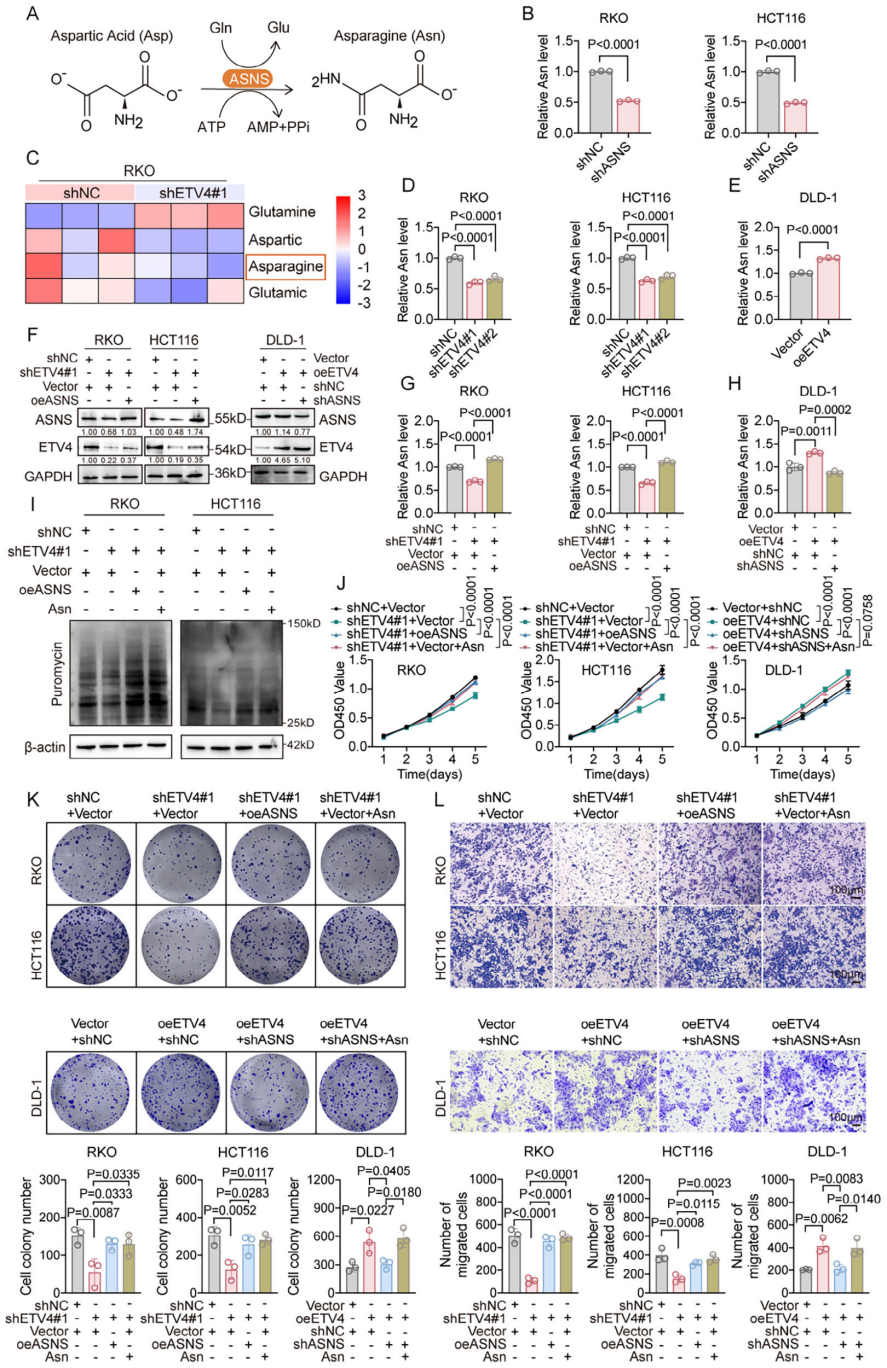
ETS variant transcription factor 4 (ETV4) promotes colorectal cancer (CRC) cell proliferation and migration by regulating asparagine synthetase (ASNS)‐dependent asparagine biosynthesis. (A) Schematic of the de novo asparagine (Asn) synthesis pathway catalyzed by the enzyme ASNS. (B) Intracellular Asn levels in RKO and HCT116 cells, confirming the efficacy of ASNS knockdown (*n* = 3). (C) Targeted metabolomics analysis revealing reduced Asn abundance in RKO cells following ETV4 knockdown (*n* = 3). (D,E) Quantification of intracellular Asn levels showing a decrease upon ETV4 knockdown in RKO and HCT116 cells (D) and an increase upon ETV4 overexpression in DLD‐1 cells (E) (*n* = 3). (F) Western blot confirming successful ASNS re‐expression in ETV4‐knockdown cells and ASNS knockdown in ETV4‐overexpressing cells. (G,H) Quantification of Asn levels showing that ASNS re‐expression restored Asn levels in ETV4‐knockdown cells (G), while ASNS knockdown suppressed the elevated Asn levels in ETV4‐overexpressing cells (H) (*n* = 3). (I) Puromycin incorporation assay measuring nascent protein synthesis. (J–L) The ETV4‐depletion‐induced defects in malignant phenotypes were reversed by either ASNS re‐expression or exogenous Asn (0.1 mM) supplementation. This was evidenced by restored cell proliferation (CCK‐8 assay; J), clonogenicity (colony formation; K), and migration (Transwell assay; L). Scale bar, 100 µm (L). Quantification for (K) and (L) is shown below the representative images (*n* = 3). Data are presented as mean ± SD (B,D,E,G,H,J–L). Statistical significance was determined by two‐tailed unpaired Student's *t*‐test (B,E), one‐way ANOVA with Dunnett's multiple comparisons test (D), one‐way ANOVA with Tukey's multiple comparisons test (G,H,K,L), or two‐way ANOVA with Tukey's multiple comparisons test (J). *p* values are provided in the figure, and *p* < 0.05 was considered statistically significant.

Given that ASNS emerged as a direct downstream target of ETV4, we next examined whether ETV4 modulates Asn metabolism in CRC cells. Asn is synthesized de novo by ASNS from aspartic acid (Asp) and glutamine (Gln) in an adenosine triphosphate (ATP)‐dependent reaction (Figure 4A). ASNS knockdown led to a marked reduction in intracellular Asn levels in RKO and HCT116 cells (Figure 4B), while ETV4 silencing similarly decreased Asn abundance, as confirmed by targeted metabolomics analysis (Figure 4C,D). Conversely, ETV4 overexpression increased Asn levels in DLD‐1 cells (Figure 4E). Western blot confirmed that ETV4 positively regulates ASNS protein expression, and that ASNS restoration rescues its loss in ETV4‐silenced cells, whereas ASNS knockdown negates the effect of ETV4 overexpression (Figure 4F).

Rescue experiments further showed that reduced Asn levels and impaired nascent protein synthesis caused by ETV4 knockdown could be partially restored by either ASNS re‐expression or exogenous Asn supplementation (Figure 4G–I). Functionally, ETV4 depletion suppressed CRC cell proliferation, clonogenic potential, and migration, effects that were rescued by ASNS re‐expression or Asn supplementation (Figure 4J–L). Together, these results demonstrate that ETV4 promotes CRC malignant phenotypes, at least in part, by transcriptionally upregulating ASNS and promoting Asn biosynthesis.

### 2.5 HGF/MET Signaling Activates ETV4 Expression via an ERK1/2–p65‐Dependent Mechanism, Forming a Positive Feedback Loop

**FIGURE 5:**
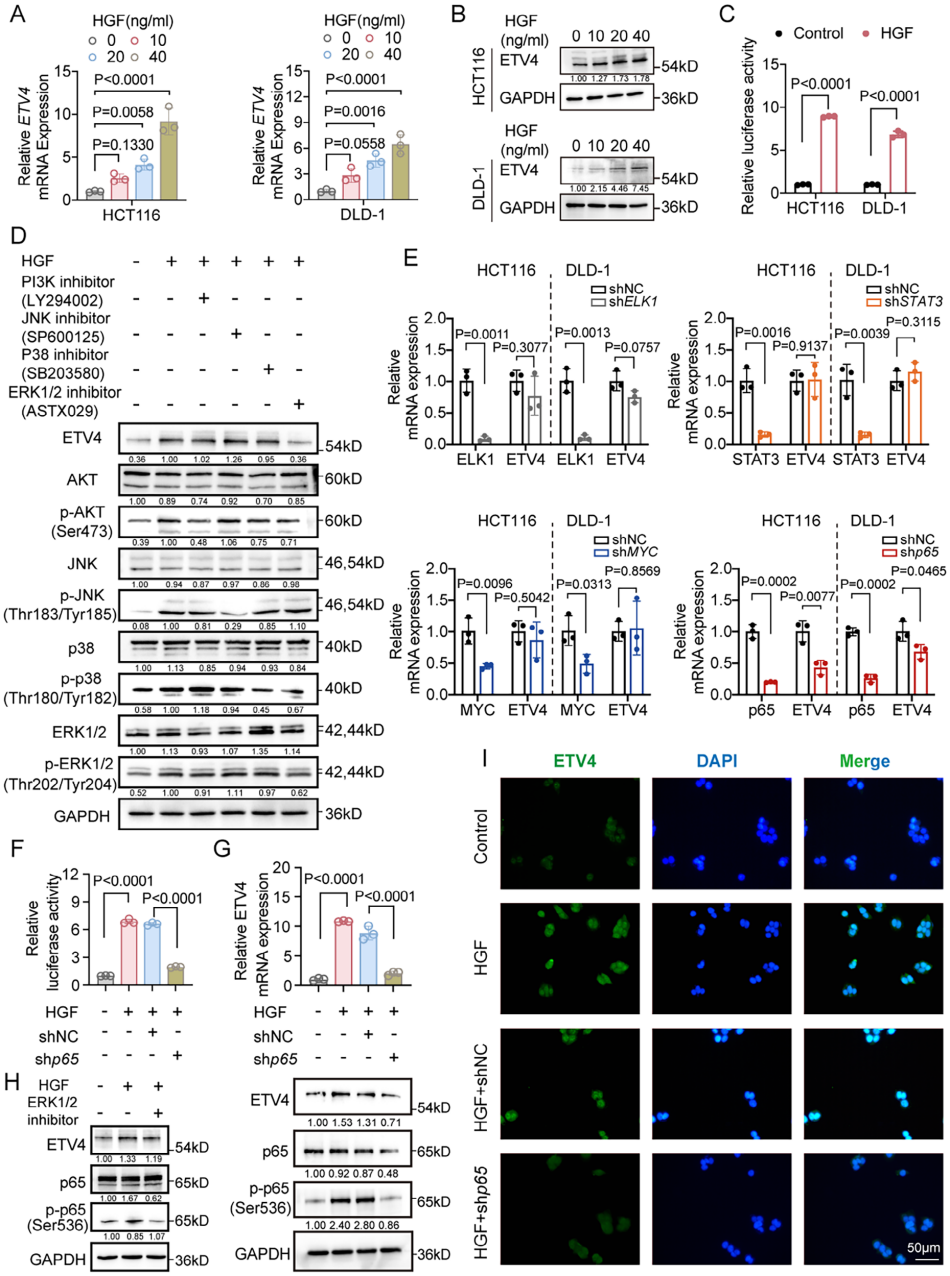
HGF/MET signaling activates ETV4 expression via an ERK1/2–p65‐dependent mechanism. (A,B) ETV4 mRNA (A, *n* = 3) and protein (B) levels in HCT116 and DLD‐1 cells treated with increasing concentrations of HGF (0, 10, 20, 40 ng/mL) for 24 h. (C) ETV4 promoter activity in HCT116 and DLD‐1 cells following HGF stimulation (40 ng/mL, 24 h), as measured by dual‐luciferase reporter assay (*n* = 3). (D) The protein levels of total and phosphorylated ERK1/2, JNK, AKT, p38, as well as ETV4 expression, were examined by Western blot in DLD‐1 cells upon HGF stimulation and corresponding inhibitor treatment. (E) Relative ETV4 mRNA expression in HCT116 and DLD‐1 cells after shRNA‐mediated knockdown of ELK1, STAT3, MYC, or RELA (p65), as measured by reverse transcription quantitative PCR (RT‐qPCR) (*n* = 3). (F) Luciferase activity of the ETV4 promoter in HCT116 cells under four conditions: (1) untransfected without HGF, (2) untransfected with HGF, (3) shNC‐transfected with HGF, and (4) shRELA‐transfected with HGF (*n* = 3). (G) RT‐qPCR (*n* = 3) and Western blot analysis of ETV4 mRNA and protein levels, respectively, in HCT116 cells subjected to the same treatments as described in (F). (H) ETV4 and total and phosphorylated p65 protein levels were assessed by Western blot analysis following HGF treatment in the presence or absence of the ERK1/2 inhibitor ASTX029. (I) Immunofluorescence analysis of ETV4 nuclear localization (green) in HCT116 cells treated under the same conditions as in (F). Nuclei were counterstained with DAPI (blue). Scale bar, 50 µm. Data are presented as mean ± SD (A,C,E–G). Statistical significance was determined by two‐tailed unpaired Student's *t*‐test (C,E), one‐way ANOVA with Dunnett's multiple comparisons test (A), or one‐way ANOVA with Tukey's multiple comparisons test (F,G). *p* values are provided in the figure, and *p* < 0.05 was considered statistically significant.

To investigate the upstream regulatory mechanism of ETV4 expression in CRC, we focused on HGF, the sole ligand for the MET RTK. While previous studies have suggested that HGF/MET signaling may influence ETV4 expression in certain cancer types [18], the regulatory relationship and underlying mechanisms in CRC remain largely unclear. In addition, our study (Figure 3) identified MET as a direct transcriptional target of ETV4, raising the possibility of a feedback regulatory circuit involving HGF and ETV4 in CRC. To address this, we treated CRC cell lines (HCT116 and DLD‐1) with recombinant HGF and observed a dose‐dependent increase in both ETV4 mRNA and protein levels (Figure 5A,B), as well as a significant enhancement of ETV4 promoter activity as measured by dual‐luciferase reporter assay (Figure 5C). Functionally, HGF stimulation promoted CRC cell proliferation and migration (Figure S5A,B), effects that were blocked by the anti‐MET antibody Onartuzumab.

To elucidate the downstream pathways mediating this effect, we employed selective pharmacological inhibitors of key MET‐activated signaling cascades, including PI3K/AKT, JNK, p38 MAPK, and ERK1/2 [19, 20, 21, 22]. Among these, only ERK1/2 inhibition by ASTX029 specifically abolished HGF‐induced ETV4 upregulation, while inhibition of PI3K, JNK, or p38 showed minimal effects (Figure 5D). Since ERK1/2 can activate several transcription factors such as ELK1, NF‐κB p65/RelA (p65), STAT3, and MYC (Figure S11) [23, 24, 25, 26], we systematically knocked down each in HCT116 and DLD‐1 cells. Only p65 knockdown resulted in a marked decrease in ETV4 mRNA (Figure 5E), implicating NF‐κB p65 as the key downstream effector.

Dual‐luciferase reporter assays confirmed that p65 silencing suppressed HGF‐induced ETV4 promoter activation (Figure 5F). Western blot analysis further demonstrated that HGF stimulation increased phosphorylation of both ERK1/2 and p65, together with elevated ETV4 protein levels. Either ERK1/2 inhibition with ASTX029 or MET blockade reduced ERK1/2 and p65 phosphorylation and suppressed ETV4 expression (Figure 5H; Figure S5C). Additionally, p65 knockdown under HGF stimulation led to decreased total and phosphorylated p65, as well as diminished ETV4 protein levels (Figure 5G). Immunofluorescence (IF) staining corroborated these findings by showing reduced nuclear ETV4 following p65 knockdown in HGF‐treated cells (Figure 5I). Collectively, these data indicate that HGF/MET signaling activates ETV4 expression via an ERK1/2–p65 signaling axis, establishing a positive feedback loop.

### 2.6 Asparagine Promotes the Inflammatory Cancer‐Associated Fibroblast (iCAF) Like Activation of Hepatic Stellate Cells (HSCs) in the Metastatic Colorectal Cancer Microenvironment

To investigate the cellular source and regulatory mechanisms of HGF in the CRC microenvironment, we first analyzed a published scRNA‐seq dataset of primary CRC (EMTAB8107). The analysis confirmed that fibroblasts are the primary source of HGF in CRC tissues, consistent with previous reports underscoring the central role of fibroblast‐derived HGF in tumor progression (Figure S7A,B) [7]. However, within the critical microenvironment of CRC liver metastases (CRLM), the specific subtypes, tissue of origin, and upstream regulatory signals for these HGF‐secreting fibroblasts remain undefined.

**FIGURE 6:**
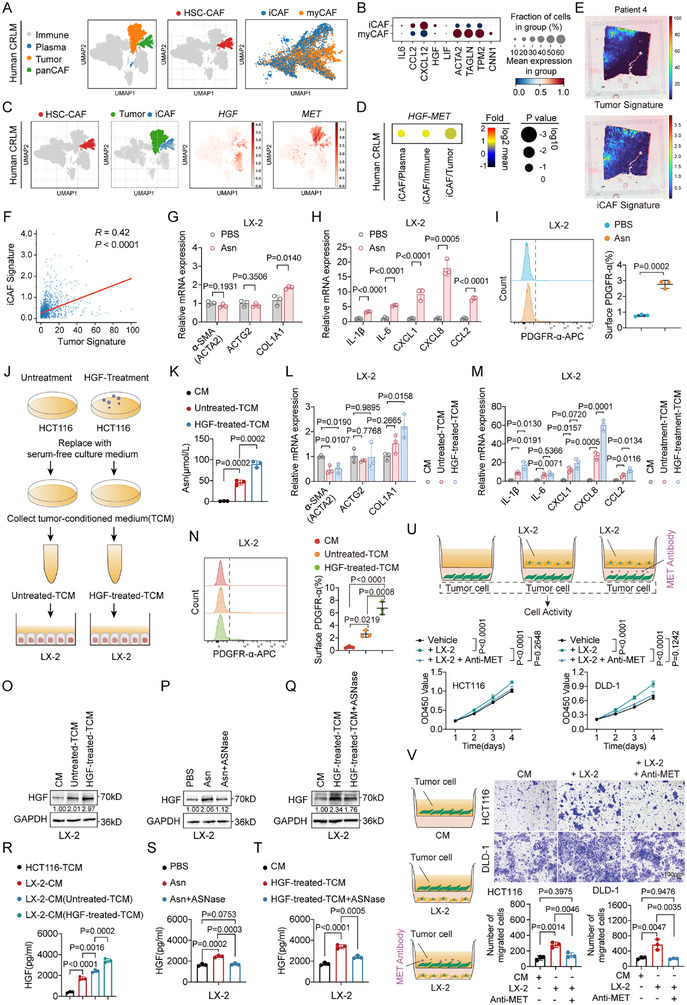
A hepatocyte growth factor (HGF)‐driven metabolic switch in colorectal cancer (CRC) cells induces an inflammatory cancer‐associated fibroblast (iCAF)‐associated inflammatory signature in hepatic stellate cells (HSCs) via asparagine (Asn). (A) Uniform Manifold Approximation and Projection (UMAP) projection of scRNA‐seq data from human CRC liver metastases (CRLM) tissues, showing the major identified cell subpopulations (left) and the distribution of HSC‐derived CAFs (HSC‐CAFs), iCAFs, and myofibroblastic CAFs (myCAFs) within the panCAF population (right). (B) Dot plot showing the expression levels and percentage of cells expressing key marker genes for iCAF and myCAF subpopulations. (C) Feature plots illustrating the expression of HGF and its receptor MET across all cell clusters in the UMAP space. (D) Bubble plot summarizing the results of ligand‐receptor interaction analysis, highlighting the significant interaction between HGF from iCAFs and MET on tumor cells. (E,F) Abundance estimation (E) and correlation analysis (F) of tumor and iCAF signatures in spatial transcriptomics sections. The correlation was evaluated using a two‐sided Pearson correlation test. Pearson correlation coefficients (*R*) and *p* values are indicated. (G–I) Effects of Asn (0.1 mM, 48 h) on LX‐2 cell activation, including reverse transcription quantitative PCR (RT‐qPCR) analysis of myCAF‐associated markers (G) and iCAF‐associated inflammatory markers (H), and flow cytometric quantification of surface PDGFR‐α expression (I) (*n* = 3). (J) Schematic of the experimental workflow for preparing tumor‐conditioned medium (TCM) from HCT116 cells. Experimental groups: CM (culture medium control); Untreated‐TCM (from vehicle‐treated cells); and HGF‐treated‐TCM (from HGF‐treated cells). (K) Measurement of Asn concentration in the indicated conditioned media (*n* = 3). (L–N) Effects of HGF‐treated tumor‐conditioned medium (HGF‐treated‐TCM) on LX‐2 cell activation, assessed by RT‐qPCR of myCAF‐associated markers (L) and iCAF‐associated inflammatory markers (M), and flow cytometric quantification of surface PDGFR‐α expression under different TCM conditions (N) (*n* = 3). (O–Q) Western blot analysis of HGF protein expression in LX‐2 cells treated with different TCMs (O), Asn alone or combined with ASNase (P), and HGF‐treated‐TCM alone or combined with ASNase (Q). (R) ELISA quantification of HGF levels in HCT116‐TCM and in conditioned media from LX‐2 cells that were pretreated with either standard medium, untreated‐TCM, or HGF‐treated‐TCM (*n* = 3). (S,T) ELISA quantification of HGF secreted by LX‐2 cells treated with Asn (S) or HGF‐treated‐TCM (T), in the presence or absence of ASNase (*n* = 3). (U) CCK‐8 proliferation assay of HCT116 and DLD‐1 cells, which were seeded in the lower chamber and cocultured with LX‐2 cells placed in the upper Transwell inserts. The MET‐blocking antibody Onartuzumab (10 µg/mL) was added to assess the role of MET signaling (*n* = 3). (V) Transwell migration assay evaluating the chemotactic migration of HCT116 and DLD‐1 cells toward LX‐2 cells seeded in the lower chamber. Onartuzumab (10 µg/mL) was used to block MET signaling. Representative images and quantification are provided (*n* = 3). Scale bar, 100 µm. Data are presented as mean ± SD (G–I,K–N,R–V). Statistical significance was determined by two‐tailed unpaired Student's *t*‐test (G–I), one‐way ANOVA with Tukey's multiple comparisons test (K–N,R–T,V), or two‐way ANOVA with Tukey's multiple comparisons test (U). *p* values are indicated in the figure, and *p* < 0.05 was considered statistically significant.

To address this, we performed scRNA‐seq on human CRLM tissues. Based on distinct gene expression signatures, we classified fibroblasts into three major subsets: HSC‐derived CAFs (HSC‐CAFs), CAFs exhibiting an inflammatory phenotype (iCAFs, enriched for cytokines), and those with prominent myofibroblastic features (myofibroblastic cancer‐associated fibroblasts (myCAFs), characterized by contractile markers). Uniform Manifold Approximation and Projection (UMAP) projection analysis revealed that HSC‐CAFs constituted the majority of the panCAF population, with the iCAF subcluster largely residing within the HSC‐CAF population. These results suggest that HSCs may serve as a key source of CAFs in CRLM and potentially differentiate into an iCAF phenotype (Figure 6A). Gene expression profiling confirmed that iCAFs preferentially express various inflammatory mediators, including HGF, whereas myCAFs are enriched for contractile genes such as ACTA2 and TAGLN (Figure 6B). Correspondingly, HGF expression was primarily localized to the iCAF cluster, while its receptor, MET, was highly enriched in tumor cells (Figure 6C). Ligand–receptor interaction analysis identified a significant iCAF‐to‐tumor HGF‐MET paracrine signaling axis (Figure 6D). Furthermore, analysis of spatial transcriptomics data revealed a significant positive correlation between tumor and iCAF signatures in CRLM sections (Figure 6E,F). This finding suggests that iCAFs and tumor cells reside in close spatial proximity, providing the structural basis for their paracrine interactions.

Given the established role of cytokines in modulating CAF phenotypes, we first sought to confirm this in our system. Treatment with recombinant human IL‐1β or TNF‐α markedly induced the expression of the iCAF‐associated inflammatory markers (IL1B, IL6, CXCL1, CXCL8, and CCL2) in LX‐2 cells. In contrast, the expression of myCAF‐associated markers (ACTA2, ACTG2, and COL1A1) was largely unaffected (Figure S6A,C). IL‐6 had a minimal impact on either iCAF or myofibroblastic marker expression (Figure S6B). This prompted us to investigate whether metabolic cues could similarly drive this switch toward an iCAF‐like state. We hypothesized that Asn could modulate the phenotypic plasticity of HSC‐derived CAFs. Direct Asn treatment of LX‐2 cells did not significantly alter the expression of myCAF‐associated markers (Figure 6G) but induced the expression of the iCAF‐associated inflammatory markers (Figure 6H) and increased the surface expression of the iCAF activation marker PDGFR‐α (Figure 6I).

We next explored a potential feedback loop initiated by tumor cells. We generated tumor‐conditioned medium (TCM) from HCT116 cells pretreated with HGF (HGF‐treated‐TCM) to mimic the effect of CAF‐derived signals (Figure 6J). Quantitative ELISA analysis revealed that HGF stimulation led to a significant increase in the concentration of Asn in the TCM (Figure 6K). This HGF‐treated‐TCM phenocopied the effects of direct Asn stimulation on LX‐2 cells, inducing the iCAF‐associated inflammatory markers and PDGFR‐α (Figure 6M,N) without significantly affecting myCAF‐associated markers (Figure 6L).

Western blot and ELISA analyses confirmed that LX‐2 cells secreted significantly more HGF when stimulated with either HGF‐treated‐TCM or direct Asn. The pro‐secretory effect of Asn was abrogated by l‐asparaginase (ASNase), confirming that Asn is a direct upstream driver of HGF production. Similarly, the HGF secretion induced by the Asn‐rich HGF‐treated‐TCM was reversed by ASNase treatment (Figure 6O–T). Finally, we investigated the functional consequences of this HSC‐derived HGF. The proliferation and migration of both HCT116 and DLD‐1 cells were significantly enhanced by indirect coculture with LX‐2 cells (Figure 6U,V). These malignant phenotypes were attenuated by Onartuzumab, a MET‐blocking antibody, confirming that the observed phenotypes are mediated by the canonical HGF‐MET signaling axis established in our single‐cell analysis. Collectively, these findings uncover a novel mechanism of stromal reprogramming, in which tumor‐derived Asn functions as a metabolic signal to direct HSCs toward a pro‐metastatic, HGF‐secreting iCAF‐like state.

### 2.7 Asparagine Reprograms Patient‐Derived CAFs Toward an Inflammatory, HGF‐Secreting State to Promote CRC Growth

**FIGURE 7:**
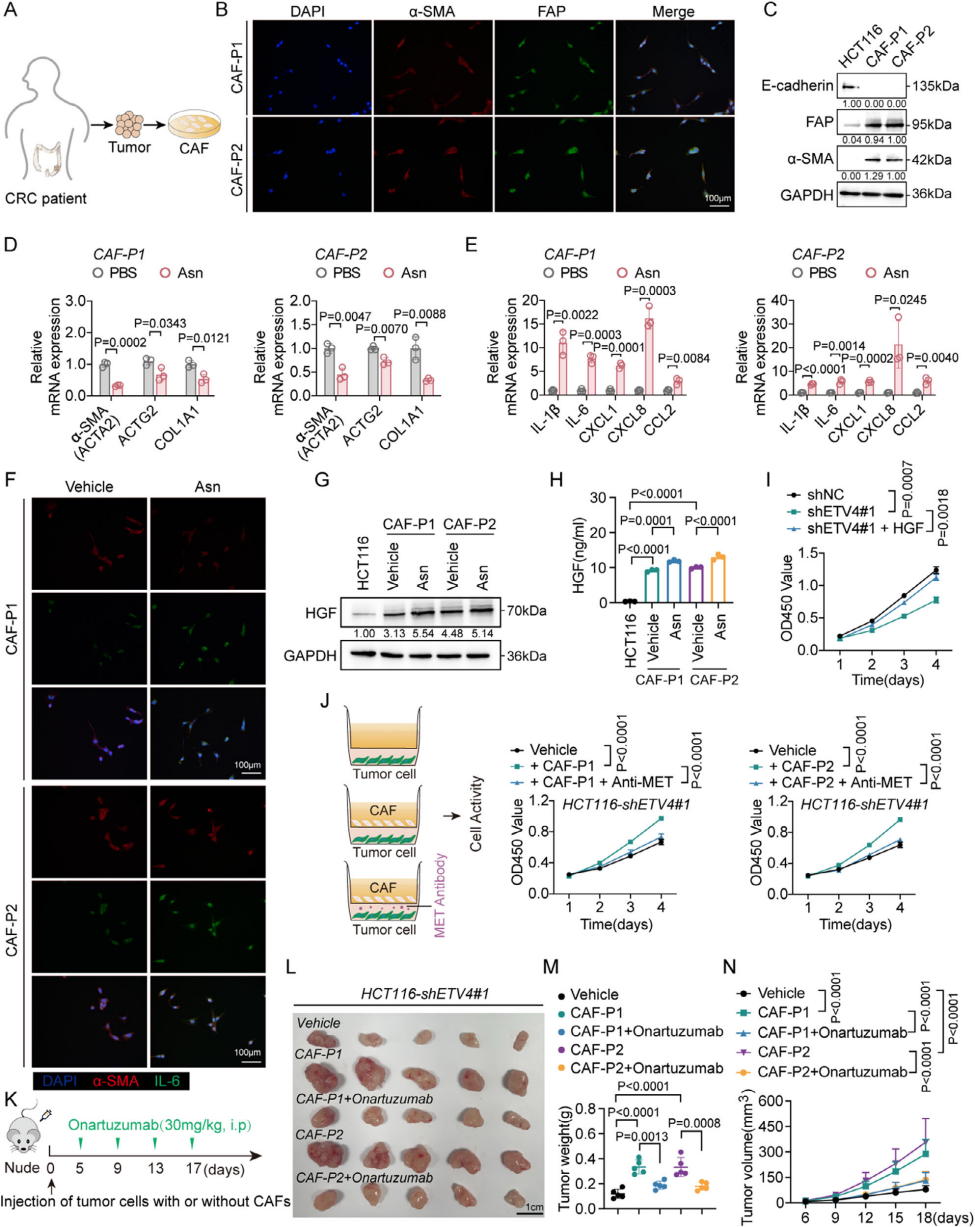
Asparagine (Asn) drives an inflammatory cancer‐associated fibroblast (iCAF) program in patient‐derived CAFs to support colorectal cancer (CRC) growth. (A) Schematic of primary CAF isolation from human CRC tissues. (B) Immunofluorescence staining of primary CAFs (CAF‐P1/P2) showing the expression of α‐SMA (red), FAP (green), and DAPI (blue). DAPI was used to stain the nuclei. Scale bar, 100 µm. (C) Western blot analysis of E‐cadherin, FAP, and α‐SMA expression in HCT116 cells and primary CAFs. (D,E) Reverse transcription quantitative PCR (RT‐qPCR) analysis of myofibroblastic (D) and inflammatory (E) marker gene expression in CAFs treated with vehicle (PBS) or Asn (0.1 mM, 48 h) (*n* = 3). (F) Immunofluorescence images of α‐SMA (red) and IL‐6 (green) staining in CAFs treated with vehicle or Asn (0.1 mM, 48 h) (*n* = 3). DAPI (blue) was used to stain the nuclei. Scale bar, 100 µm. (G,H) Western blot (G) and ELISA (H, *n* = 3) analysis of HGF protein expression and secretion in HCT116 cells and CAFs treated with vehicle or Asn (0.1 mM, 48 h). (I) CCK‐8 proliferation assay of HCT116‐shETV4#1 treated with or without recombinant HGF (40 ng/mL) (*n* = 3). (J) CCK‐8 proliferation assay of HCT116‐shETV4#1 cocultured with CAFs in the presence or absence of Onartuzumab (10 µg/mL) (*n* = 3). (K) Schematic of the in vivo coinjection model. (L–N) Gross images (scale bar, 1 cm) (L), tumor weights (M), and growth curves (N) of xenograft tumors from mice injected with HCT116‐shETV4#1 alone, or coinjected with CAFs (CAF‐P1/P2) in the presence or absence of Onartuzumab (30 mg/kg) treatment (*n* = 5 mice per group). Data are presented as mean ± SD (D,E,H–J,M,N). Statistical significance was determined by two‐tailed unpaired Student's *t*‐test (D,E), one‐way ANOVA with Tukey's multiple comparisons test (H,M), or two‐way ANOVA with Tukey's multiple comparisons test (I,J,N). *p* values are indicated in the figure, and *p* < 0.05 was considered statistically significant.

The immortalized LX‐2 cell line may not fully recapitulate the plasticity of primary stromal cells. To validate our findings in a more clinically relevant system, we isolated primary CAFs from CRC tissues of two independent patients (Figure 7A). Immunofluorescence and Western blot analyses confirmed these patient‐derived CAFs (CAF‐P1 and CAF‐P2) were α‐SMA^+^/FAP^+^/E‐cadherin^−^, indicative of a predominantly myofibroblastic CAF (myCAF) baseline phenotype (Figure 7B,C).

We next investigated whether Asn could drive a phenotypic switch in primary CAFs. Notably, Asn treatment downregulated myCAF‐associated markers (ACTA2, ACTG2, and COL1A1) (Figure 7D) while upregulating iCAF‐associated inflammatory markers (IL1B, IL6, CXCL1, CXCL8, and CCL2) (Figure 7E). Immunofluorescence staining revealed that Asn‐treated CAFs exhibited reduced α‐SMA accompanied by upregulation of the cytokine IL‐6 (Figure 7F). Furthermore, Western blot and ELISA analyses indicated that Asn stimulation significantly increased intracellular HGF protein expression and extracellular secretion in both CAF‐P1 and CAF‐P2 (Figure 7G,H). Functionally, recombinant HGF effectively rescued the proliferation defects induced by ETV4 knockdown (Figure 7I), while pharmacological blockade of MET signaling using Onartuzumab abrogated the growth‐promoting effects of CAF coculture in vitro (Figure 7J). To validate this crosstalk in vivo, we employed a coinjection xenograft model. While ETV4‐silenced HCT116 cells formed small tumors when injected alone, coinjection with primary CAFs (CAF‐P1 or CAF‐P2) significantly restored tumor growth. This growth advantage conferred by CAFs was attenuated by systemic treatment with Onartuzumab (Figure 7K–N). Collectively, these findings demonstrate that Asn reprograms patient‐derived CAFs toward an inflammatory, HGF‐secreting state that reciprocally promotes CRC growth through HGF/MET signaling.

### 2.8 Combined Targeting of HGF/MET Signaling and Asparagine Metabolism Suppresses CRC Growth and Metastasis In Vivo

**FIGURE 8:**
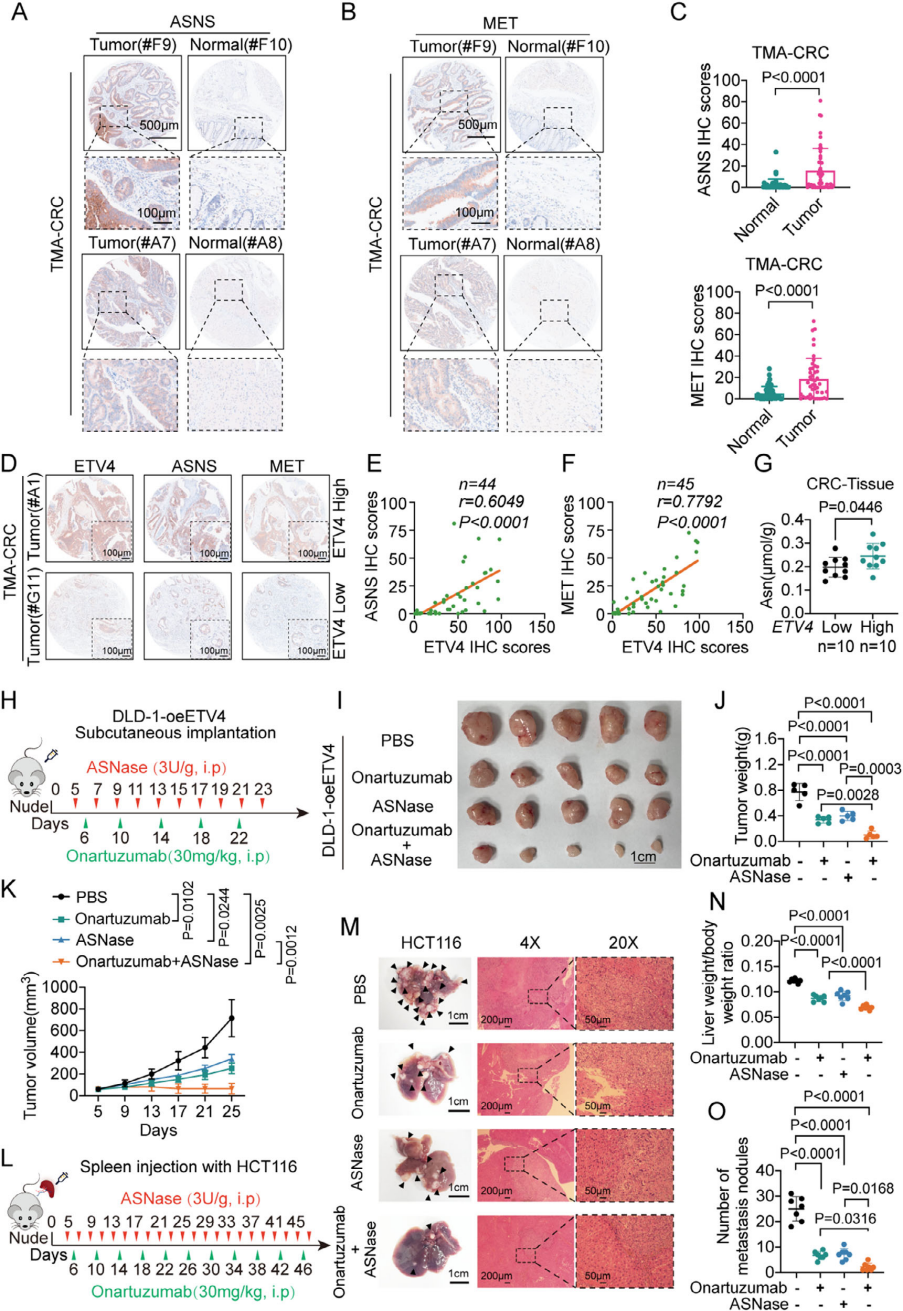
Combined MET blockade with Onartuzumab and asparagine (Asn) deprivation suppresses colorectal cancer (CRC) growth and metastasis in vivo. (A,B) Representative immunohistochemistry (IHC) staining of asparagine synthetase (ASNS) (A) and MET (B) in paired human CRC tumor and adjacent normal tissues. Scale bars, 500 µm (main images) and 100 µm (insets). (C) Quantification of IHC scores for ASNS (*n* = 46) and MET (*n* = 45) in paired tissues. (D) Representative IHC images showing ASNS and MET staining in tumors with low versus high ETV4 expression. Scale bar, 100 µm. (E,F) Correlation plots of IHC scores for ASNS vs. ETV4 (E, *n* = 44) and MET vs. ETV4 (F, *n* = 45) in the CRC tissue microarray (TMA). Pearson correlation coefficients (*r*) and *p* values are indicated. (G) Intratumoral Asn levels in ETV4‐high and ETV4‐low CRC tissues (*n* = 10 per group). (H) Schematic of the subcutaneous xenograft model using DLD‐1‐oeETV4 cells treated with vehicle (PBS), Onartuzumab (anti‐MET antibody), ASNase, or combination therapy. (I–K) Images of subcutaneous tumors (I, scale bar, 1 cm), final tumor weights (J), and tumor growth curves (K) for the different treatment groups (*n* = 5 mice per group). (L) Schematic of the liver metastasis model using HCT116 cells, with treatment groups identical to those in (H). (M–O) Analysis of liver metastasis, including representative liver images and hematoxylin and eosin (H&E) staining (M), liver‐to‐body weight ratio (N), and number of metastatic nodules (O) (*n* = 6 mice per group). Scale bars, 1 cm (gross image), 200 µm (4×), and 50 µm (20×) (M). Data are presented as mean ± SD (C,G,J,K,N,O). Statistical significance was determined by two‐tailed paired Student's *t*‐test (C,G), two‐sided Pearson correlation analysis (E,F), one‐way ANOVA with Tukey's multiple comparisons test (J,N,O), or two‐way ANOVA with Tukey's multiple comparisons test (K). *p* values are indicated in the figure, and *p* < 0.05 was considered statistically significant.

The codependency on the HGF/MET and ASNS pathways suggests a therapeutic vulnerability. We first assessed the clinical relevance of this axis in human CRC tissues. IHC staining of tissue microarrays (TMAs) showed that ASNS and MET were significantly upregulated in tumors compared to adjacent normal tissues (Figure 8A–C). Moreover, strong positive correlations were observed between the expression of ETV4 and that of both ASNS and MET (Figure 8D–F). Consistently, intratumoral Asn levels were significantly elevated in ETV4‐high CRC tissues (Figure 8G).

These findings were validated in xenograft models using CRC cells with manipulated ETV4 expression, where ETV4 knockdown in HCT116 cells reduced ASNS and MET protein levels, while ETV4 overexpression in DLD‐1 cells increased their expression (Figure S8A,B). Analysis of TCGA data confirmed that ASNS and MET mRNA levels were significantly higher in the TCGA‐COAD, ‐READ, and combined CRC cohorts compared to normal tissues (Figure S8C–E). Furthermore, this upregulation in tumors versus adjacent normal tissues was consistently validated across multiple GEO datasets using paired samples (Figure S8F). High ASNS or MET expression was associated with poorer overall survival in CRC patients (ASNS: hazard ratio [HR] = 1.29, *p* = 0.014; MET: HR = 1.44, *p* = 0.0042) (Figure S8G). Clinicopathological analysis revealed that higher ASNS expression was associated with distant metastasis (M1 stage, *p* = 0.034), while higher MET expression showed a strong trend toward association with M1 stage (*p* = 0.071) (Tables S7 and S8). Consistent with their role in tumor progression, single‐cell data confirmed that ASNS and MET are primarily expressed by malignant epithelial cells (Figure S7C–F).

Based on this rationale, we evaluated a dual‐targeting strategy in vivo. In a subcutaneous xenograft model using DLD‐1 cells overexpressing ETV4, combination therapy with the anti‐MET antibody Onartuzumab and ASNase resulted in significantly greater tumor growth inhibition than either monotherapy (Figure 8H–K). In a liver metastasis model, the combination therapy markedly reduced the number of hepatic metastatic nodules and decreased the liver‐to‐body weight ratio (Figure 8L–O). Collectively, these results demonstrate that cotargeting the HGF/MET and Asn metabolic pathways is an effective therapeutic strategy that disrupts tumor‐intrinsic growth signals and the metastatic niche, offering a promising approach for the treatment of advanced CRC.

## 3 Discussion

Our study identifies a previously undefined oncogenic network in CRC, positioning the transcription factor ETV4 at the core of a dynamic interplay linking signal amplification, metabolic reprogramming, and stromal microenvironment crosstalk to drive CRC growth and liver metastasis. By demonstrating that ETV4 links HGF/MET signaling with Asn metabolism, we identify a metabolite‐driven, bidirectional communication axis between tumor cells and HSCs as well as CAFs, providing mechanistic insight into how oncogenic signaling and metabolic outputs cooperatively shape a pro‐tumorigenic microenvironment.

The HGF/MET signaling pathway is a well‐established driver of malignancy in CRC, where its hyperactivation is correlated with invasion, therapeutic resistance, and poor patient prognosis [27, 28, 29]. Consequently, it has become a potential therapeutic target, with several MET inhibitors under clinical investigation [30, 31]. However, their efficacy as monotherapies is frequently undermined by intrinsic or acquired resistance, a common challenge for targeted therapies against RTKs [13]. Our discovery of an HGF/MET–ETV4–MET positive feedback loop offers a potential molecular explanation for this phenomenon. Such feedback mechanisms, which confer signaling autonomy, are potent drivers of tumorigenesis and drug resistance, as exemplified by autocrine loops involving EGFR in lung cancer or FGFR in liver cancer [18, 32]. Our work establishes a similar paradigm for the HGF/MET axis, where ETV4, a known downstream effector of RAS–MAPK signaling [33], not only responds to HGF but also transcriptionally upregulates MET. This circuit likely renders cancer cells hyper‐responsive to ambient HGF in the liver microenvironment and provides a robust mechanism for sustaining MET signaling even under therapeutic pressure, highlighting the necessity of cotargeting multiple nodes within this circuit [34, 35, 36].

Our work bridges this oncogenic signaling pathway with a critical metabolic dependency. The reprogramming of metabolism is a core hallmark of cancer [14], with amino acid metabolism emerging as a particularly crucial node for therapeutic intervention [37]. The nonessential amino acid Asn has recently gained prominence since the landmark study by Knott et al. revealed that limiting its bioavailability suppresses breast cancer metastasis [15]. Subsequent research has corroborated the importance of Asn and its synthesizing enzyme, ASNS, in other malignancies, including leukemia, osteosarcoma, and pancreatic cancer, where it supports protein synthesis, redox balance, and survival under nutrient deprivation [16, 38, 39]. Our study advances this field by identifying a previously unrecognized upstream regulatory mechanism for ASNS overexpression in CRC, establishing a direct linear cascade from HGF/MET activation to ETV4‐mediated ASNS transcription. This finding directly links a classical oncogenic driver to a key metabolic vulnerability, clarifying the question of how cancer cells orchestrate the upregulation of this crucial metabolic enzyme.

We further identify the function of tumor‐derived Asn as an extracellular signaling molecule—a “metabokine”—that actively modulates the metastatic niche. The concept that metabolites can act as signaling agents to mediate intercellular communication is a rapidly advancing field, with lactate, succinate, and arginine being recognized for their roles in modulating immune and stromal cell functions. Our work adds Asn to this growing list of signaling metabolites [40, 41, 42]. We demonstrate that Asn secreted by CRC cells specifically induces HSCs, the principal precursors of CAFs in the liver [7], to adopt an iCAF‐like phenotype. This is particularly significant in the context of CAF heterogeneity, a feature of the TME with profound functional consequences. The iCAF subtype, characterized by inflammatory cytokine secretion, has been shown to promote chemoresistance and an immunosuppressive environment, contrasting with myCAFs that are more involved in matrix deposition [5, 7]. Notably, while Asn treatment in LX‐2 cells robustly induced iCAF‐associated inflammatory markers, it did not significantly suppress myofibroblastic markers. This contrasts with our observations in patient‐derived CAFs, where a negative correlation between these signatures is observed. This discrepancy likely reflects the limited plasticity of the immortalized LX‐2 cell line compared to primary cells, which retain the capacity for dynamic lineage reprogramming. Nevertheless, the LX‐2 model allows us to specifically isolate the gain‐of‐function aspect of iCAF‐like activation, confirming that Asn is sufficient to drive the prometastatic secretory program. Our findings provide a novel metabolic determinant for this phenotypic switch, demonstrating how tumor cells can metabolically reprogram the hepatic microenvironment by inducing resident HSCs to become potent suppliers of HGF, a key factor for liver regeneration and tumorigenesis [12, 43]. thereby creating a permissive niche for their own expansion.

This detailed mechanistic understanding provides a strong rationale for the combination therapy we propose. Targeting both the HGF/MET axis and Asn metabolism simultaneously attacks the intrinsic oncogenic and metabolic support system that modulates the microenvironment. This dual‐pronged strategy aligns with the broader trend in oncology toward developing synergistic combination therapies to overcome resistance and improve efficacy. While l‐asparaginase (ASNase) is a key component of treatment for acute lymphoblastic leukemia [16], its use in solid tumors has been limited, partly by immunogenicity and poor tumor penetration [44, 45]. Newer generation, less immunogenic asparaginases, or small‐molecule ASNS inhibitors [46], combined with potent MET‐directed therapies, such as tyrosine kinase inhibitors (TKIs) and monoclonal antibodies [47], may enhance therapeutic efficacy. Furthermore, the ETV4–ASNS/MET expression signature could serve as a robust biomarker panel to stratify CRC patients who are most likely to respond to this targeted combination therapy.

Our study has limitations. The preclinical validation primarily utilized the splenic injection model; while reproducible, this approach does not recapitulate the initial spontaneous invasion steps of the metastatic cascade. Validation in more sophisticated systems, such as orthotopic cecal implantation models that recapitulate spontaneous liver metastasis or PDO‐HSC coculture systems, is therefore warranted to dissect the spatiotemporal evolution of metastasis [48, 49]. Additionally, although we confirmed the phenotype in immunocompetent mice, the specific immunomodulatory mechanisms of the ETV4‐Asn axis require deeper profiling. Beyond preclinical models, prospective validation in larger, multicenter clinical cohorts is needed to firmly establish the prognostic value of the ETV4–ASNS/MET signature, and to further evaluate whether the intratumoral Asn metabolic status may serve as a potential biomarker for predicting responsiveness to MET‐targeted therapies or metabolic interventions. On a mechanistic level, the precise machinery mediating Asn uptake in HSCs or CAFs remains to be characterized, likely involving SLC family transporters such as SLC1A5 or SLC6A12 [50, 51].

**FIGURE 9:**
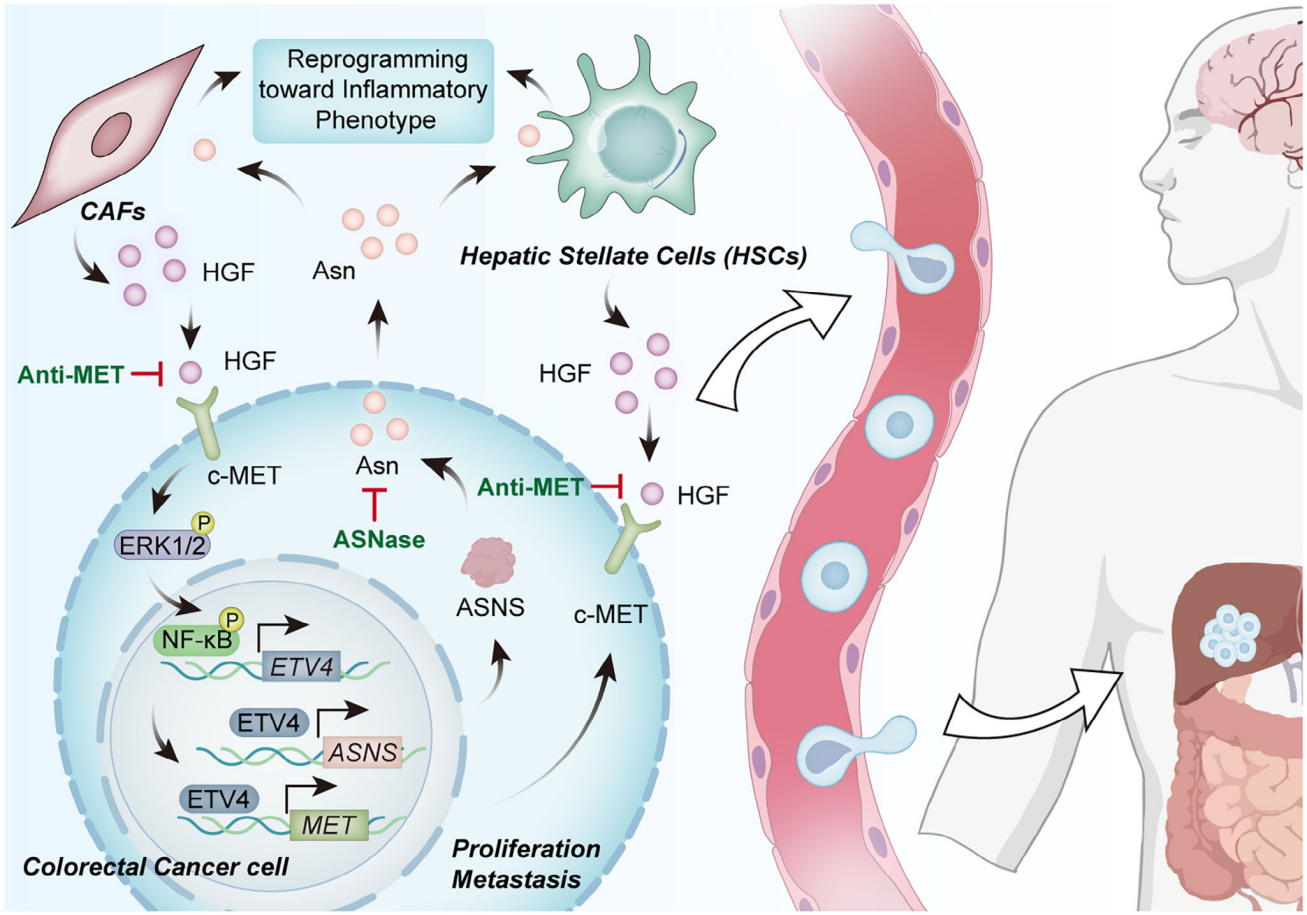
Schematic model of the ETV4‐centric regulatory network in colorectal cancer (CRC). ETV4 coordinates a dual transcriptional program by upregulating MET and asparagine synthetase (ASNS). The resulting asparagine (Asn) secretion acts as a metabolic signal, reprogramming hepatic stellate cells (HSCs) and primary cancer‐associated fibroblasts (CAFs) toward an inflammatory phenotype. These activated stromal cells release hepatocyte growth factor (HGF) to sustain the MET–ERK1/2–NF‐κB–ETV4 axis, establishing a positive feedback loop that promotes CRC growth and liver metastasis.

In conclusion, our research places ETV4 at the center of a regulatory hub that connects oncogenic signaling, metabolic reprogramming, and microenvironment remodeling. The discovery of the self‐sustaining “HGF/MET → ETV4 → ASNS → Asparagine → iCAFs and iCAF‐like HSCs → HGF” circuit provides a mechanistic framework for understanding CRC liver metastasis, offering a rational basis for a combination therapy designed to disrupt this synergistic network (Figure 9).

## 4 Experimental Section

### 4.1 Ethics Statement

All human tissue samples were collected with informed consent and approved by the Institutional Research Ethics Committee of Zhongnan Hospital of Wuhan University (Approval No. 2020110) for research purposes. CRC specimens were obtained from patients at Zhongnan Hospital of Wuhan University (Wuhan, China) following pathological diagnosis. Commercially available TMAs were purchased from Shanghai Weiao Biotechnology Co., Ltd., in compliance with donor informed consent and ethical regulations.

### 4.2 Human Samples

Three pairs of CRC lesions and matched adjacent noncancerous tissues were obtained from CRC patients at Zhongnan Hospital of Wuhan University and subjected to RNA‐seq. Additionally, 20 primary CRC tissue samples were collected from the same hospital for ETV4 mRNA quantification and the measurement of intratumoral Asn content. TMAs containing 48 paired CRC and adjacent normal tissues were obtained from Shanghai Weiao Biotechnology Co., Ltd., for the evaluation of ETV4, ASNS, and MET expression and their correlations in CRC tissues. Due to partial tissue detachment or suboptimal staining quality, the final numbers of evaluable tissue pairs for ETV4, ASNS, and MET were 45, 46, and 45, respectively. All tissue samples were collected with the informed consent of the patients, and the Institutional Review Board approved this study.

### 4.3 Cell Culture and Reagents

Human CRC cell lines SW620 (RRID: CVCL_0547), DLD‐1 (RRID: CVCL_0248), HCT116 (RRID: CVCL_0291), LoVo (RRID: CVCL_0399), and RKO (RRID: CVCL_0504) were obtained from the American Type Culture Collection (ATCC, Manassas, VA, USA). The normal human colonic epithelial cell line NCM460 (RRID: CVCL_0460), the immortalized human HSC line LX‐2 (RRID: CVCL_5792), the human embryonic kidney cell line 293T (RRID: CVCL_0063), and the mouse colon cancer cell line MC38 (RRID: CVCL_B288) were purchased from the Cell Bank of the Chinese Academy of Sciences (Shanghai, China). All cell lines were authenticated by short tandem repeat (STR) profiling and regularly confirmed to be mycoplasma‐free. All cell lines were maintained in Dulbecco's Modified Eagle Medium (DMEM, Gibco, Cat. #11965092) supplemented with 10% fetal bovine serum (FBS, Gibco, Cat. #10270106) and 1% penicillin‐streptomycin (Gibco, Cat. #15140122). For experiments requiring the measurement of extra‐ and intracellular Asn, cells were cultured in Asn‐free DMEM (Servicebio, Wuhan, China, Cat. #G4524) supplemented with 10% dialyzed FBS (dFBS, Gibco, Cat. #26400044) and 1% penicillin‐streptomycin. All cells were cultured at 37°C in a humidified atmosphere containing 5% CO_2_.

The following reagents were purchased from MedChemExpress (MCE, Monmouth Junction, NJ, USA): recombinant human proteins HGF (Cat. #HY‐P70627), IL‐1β (Cat. #HY‐P7028), IL‐6 (Cat. #HY‐P7044), and TNF‐α (Cat. #HY‐P70426A); specific signaling inhibitors LY294002 (Cat. #HY‐10108), SP600125 (Cat. #HY‐12041), SB203580 (Cat. #HY‐10256), and ASTX029 (Cat. #HY‐126288); the anti‐MET monoclonal antibody Onartuzumab (Cat. #HY‐P99250); and l‐asparaginase (ASNase, Cat. #HY‐P1923). l‐Asn (Cat. #L425606) was obtained from Aladdin (Shanghai, China).

### 4.4 Plasmid Construction and Lentiviral Transduction

Double‐stranded oligonucleotides encoding the short hairpin RNA (shRNA) sequences targeting human ETV4, ASNS, MET, and mouse ETV4 were synthesized and cloned into the double‐digested pLKO.1‐puro lentiviral vector (Addgene, Cat. #8453) with AgeI (Yeasen, Cat. #15056ES25) and EcoRI (Yeasen, Cat. #15010ES78) restriction enzymes. The target sequences for these shRNAs are listed in Table S1. For overexpression, full‐length human ASNS and ETV4 coding sequences were subcloned into the pCDH‐CMV‐MCS‐EF1‐Puro lentiviral vector (System Biosciences, Cat. #CD510B‐1). All constructs were verified by Sanger sequencing. Lentiviral vectors expressing ETV4 and ASNS (pCDH‐CMV‐MCS‐EF1‐Puro as plasmid backbone) and shRNA‐encoding lentiviral vectors were cotransfected with the packaging vectors psPAX2 (Addgene, Cat. #12260) and pMD2.G (Addgene, Cat. #12259) into 293T cells for lentivirus production. Cells were infected with the generated lentiviruses for 48 h. Puromycin (Millipore, Cat. #540411) selection (2 µg/mL) commenced 72 h post‐infection and continued for at least 5 days to establish stable cell lines.

### 4.5 RNA Extraction and Quantitative Real‐Time PCR (RT‐qPCR)

Total RNA was isolated from cells using TRIzol reagent (Accurate Biotechnology, Changsha, China, Cat. #AG21101). Reverse transcription was performed using 1 µg of total RNA with the Evo M‐MLV RT Kit (Accurate Biotechnology, Cat. #AG11706). RT‐qPCR was performed using SYBR Green Premix Pro Taq HS qPCR Kit (Accurate Biotechnology, Cat. #AG11701) on a CFX96 Touch Real‐Time PCR Detection System (Bio‐Rad, USA). Relative gene expression was calculated using the 2^−ΔΔCt^ method and normalized to the internal control GAPDH. All primer sequences are listed in Table S2.

### 4.6 Western Blot Analysis

Cells were lysed in RIPA buffer (Beyotime, Shanghai, China, Cat. #P0013B) supplemented with a protease inhibitor cocktail (Selleck, Shanghai, China, Cat. #B14001) and PMSF (Selleck, Cat. #S3025). Protein concentrations were determined using a BCA assay kit (Beyotime, Cat. #P0012). Equal amounts of protein were separated by SDS‐PAGE and transferred to 0.45 µm PVDF membranes (Merck Millipore, Cat. #IPVH00010). Membranes were blocked with 5% non‐fat milk in Tris‐buffered saline with Tween 20 (TBST) for 1 h at room temperature and then incubated with primary antibodies overnight at 4°C. After washing, membranes were incubated with horseradish peroxidase (HRP) conjugated secondary antibodies for 1 h at room temperature. Signals were visualized using an ECL substrate (Fudebio, Hangzhou, China, Cat. #FD8030) and captured with a Tanon 5200 Multi chemiluminescent imaging system (Tanon, Shanghai, China). Protein band intensities were quantified and normalized to GAPDH levels, with the resulting relative values displayed below each band. A list of antibodies used for this assay is provided in Table S3.

### 4.7 Puromycin Incorporation Assay

To assess global protein synthesis, cells were treated with 2 µg/mL puromycin for 30 min at 37°C before harvesting. Total protein was then extracted and analyzed by Western blot as described above, using an anti‐puromycin antibody (ABclonal, Wuhan, China, Cat. #A21205) to detect nascent polypeptides.

### 4.8 Cell Proliferation, Colony Formation, Migration, Invasion, and Wound Healing Assays

Cell proliferation was assessed using the Cell Counting Kit‐8 (CCK‐8) assay. CRC cells (1 × 10^3^ per well) were seeded into 96‐well plates, and absorbance at 450 nm was recorded after 2 h of incubation with the CCK‐8 reagent (DOJINDO, Kumamoto, Japan, Cat. #CK04). For the colony formation assay, CRC cells (1 × 10^3^ per well) were seeded into 6‐well plates and cultured for 2 weeks. Colonies were fixed with 4% paraformaldehyde, stained with 0.1% crystal violet, and subsequently counted. Cell migration and invasion were evaluated using Transwell chambers with 8‐µm pore filters (Corning, NY, USA, Cat. #3422). For invasion assays, the upper chambers were precoated with Matrigel (Corning, Cat. #356234). CRC cells (1 × 10^5^) suspended in serum‐free DMEM were added to the upper chambers, while the lower chambers were filled with DMEM containing 10% FBS, which served as a chemoattractant. Cells that had migrated to the lower surface were fixed with 4% paraformaldehyde and stained with 0.1% crystal violet. Images of three random fields per insert were captured under a microscope, and the cells were counted. For the wound healing assay, CRC cells (1 × 10^6^ per well) were seeded into 6‐well plates and cultured in DMEM containing 10% FBS until reaching confluence. A linear scratch was made in the center of each well, and images were captured immediately (0 h). The medium was then replaced with low‐serum DMEM (1% FBS), and the cells were cultured for an additional 48 or 72 h. The wound closure was photographed at the final time point, and the migration distance was quantified as the ratio of the healed wound width to the initial wound width at 0 h.

### 4.9 Tumor‐Conditioned Medium (TCM) Preparation and Coculture Assays

TCM was prepared by treating HCT116 cells with or without HGF (40 ng/mL) for 24 h. The medium was replaced with serum‐free DMEM (Servicebio, Cat. #G4524) and incubated for an additional 48 h. The supernatant was collected, centrifuged to remove cell debris, and filtered through a 0.22 µm sterile filter to remove residual debris and ensure sterility. The filtered supernatant was stored at −80°C as TCM for later use.

For functional assays, LX‐2 cells were preincubated for 12 h in DMEM with 2% FBS. Subsequently, they were treated for 48 h with a 1:1 mixture of TCM and DMEM containing 4% dFBS (final dFBS concentration: 2%). Control groups were treated with DMEM containing 2% dFBS without TCM to ensure consistent serum concentrations across all experimental groups. To explore the interaction between HSCs and CRC cells, CRC cells (1 × 10^5^) and LX‐2 cells (1 × 10^5^) were seeded in a 24‐well Transwell system (Corning). For the proliferation assay, CRC cells were seeded in the lower chamber (0.4 µm pore size, Cat. #3470) and LX‐2 cells in the upper insert. For the migration assay, LX‐2 cells were seeded in the lower chamber and CRC cells in the upper insert (8 µm pore size, Cat. #3422). The two cell types were cocultured for 48 h. In specific groups, an anti‐MET antibody Onartuzumab (10 µg/mL) was added to the coculture medium. After coculture, CRC cells were collected from the lower chamber for the proliferation assay or fixed, stained, and counted for the migration assay.

### 4.10 RNA‐Seq and Bioinformatics Analysis

Total RNA was extracted from three paired CRC tumors and adjacent tissues, as well as from control and ETV4‐knockdown RKO cells. Library construction and sequencing were performed by Novogene (Beijing, China). Public RNA‐seq datasets were downloaded from the GEO (https://www.ncbi.nlm.nih.gov/geo/) under accession numbers GSE22598, GSE113513, GSE74602, GSE44076, GSE41328, and GSE41568. TCGA‐COAD and TCGA‐READ cohort data were downloaded from the GDC portal (https://portal.gdc.cancer.gov/). Differential expression analysis was performed using the limma package in R (v4.2.1).

To identify ETV4 transcriptional targets, ChIP‐seq data were retrieved from ChIPBase v3.0 (ID41253, https://rnasysu.com/chipbase3/) and the ENCODE database (https://www.encodeproject.org). ChIP‐seq signal visualization was conducted using deepTools and the WashU Epigenome Browser. Survival analyses were conducted using the Kaplan–Meier Plotter (https://kmplot.com). Publicly available single‐cell RNA‐seq datasets from both primary CRC (EMTAB8107, ArrayExpress, https://www.ebi.ac.uk/arrayexpress/) and CRC liver metastases (GSE178318, GSE225857, and GSE231559) were analyzed. Raw data were processed using Cell Ranger (v6.0.0, 10× Genomics). Downstream analysis was performed using the Seurat R package (v4.1). UMAP was used for dimensionality reduction and visualization. Spatial transcriptomic raw data from GSE217414 were integrated with a single‐cell RNA‐seq reference dataset (GSE231559) using Seurat. Tumor and iCAF gene signatures were scored for each spatial spot on CRC liver metastasis sections and visualized by SpatialFeaturePlot. Spot‐level Pearson correlation analysis was performed to assess the spatial association between tumor and iCAF signatures.

### 4.11 Dual‐Luciferase Reporter Assay

The promoter regions of human ETV4, ASNS, and MET (from −2000 bp to +100 bp relative to the transcription start site) were amplified by PCR from human genomic DNA and cloned into the KpnI/XhoI sites of the pGL3‐Basic luciferase reporter vector (Promega, Madison, WI, USA). Truncated and site‐directed mutant promoter constructs of ASNS and MET were generated using specific primers (Table S4). All constructs were verified by Sanger sequencing. The pGL3 reporter vector, the pRL‐TK Renilla luciferase control vector (Promega), and either the ETV4 expression vector or the control vector were cotransfected into HCT116 cells at a ratio of 10:1:10. At 48 h post‐transfection, cells were lysed, and luciferase activity was measured using the Dual‐Luciferase Reporter Assay Kit (Vazyme, Nanjing, China, Cat. #DL101‐01) on a GloMax 20/20 luminometer (Promega).

### 4.12 Chromatin Immunoprecipitation and qPCR (ChIP‐qPCR)

ChIP assays were performed using the ChIP Chromatin Immunoprecipitation Kit (GeneCreate, Wuhan, China, Cat. #JKR23002A) following the manufacturer's instructions. Briefly, 1 × 10^7^ cells were cross‐linked with 1% formaldehyde, and 2 µg of anti‐ETV4 antibody (Proteintech, Cat. #10684‐1‐AP) or normal rabbit IgG (negative control) was used for immunoprecipitation. After reverse cross‐linking and DNA purification, qPCR was conducted. Results were expressed as a percentage of the input. Primer sequences are listed in Table S5.

### 4.13 Targeted Amino Acid Metabolomics by LC‐MS/MS

The intracellular concentrations of Asn were quantified using liquid chromatography‐tandem mass spectrometry (LC‐MS/MS). For sample preparation, approximately 1 × 10^7^ cells were harvested, rapidly frozen in liquid nitrogen, and stored at −80°C until analysis. Each sample was prepared in triplicate. Targeted amino acid metabolomic profiling was conducted using LC‐MS/MS by Novogene following their standard operating procedures for amino acid quantification. The final metabolite concentrations were calculated based on calibration curves generated from authentic standards.

### 4.14 Flow Cytometry (FACS) Analysis

Flow cytometry was performed to evaluate apoptosis in CRC cells and to assess fibroblast‐like phenotypic transition in LX‐2 cells. For apoptosis analysis, cells were stained using the Annexin V‐FITC/PI Apoptosis Detection Kit (Lianke Biosciences, Hangzhou, China, Cat. #AT101) according to the manufacturer's protocol. For fibroblast marker analysis, LX‐2 cells were stained with Alexa Fluor 647‐conjugated anti‐human PDGFR‐α antibody (Abclonal, Cat. #A22690). All fluorescence signals were acquired on a CytoFlex (Beckman, USA), and data were analyzed using FlowJo software (Tree Star).

### 4.15 Hematoxylin and Eosin (H&E) and Immunohistochemical (IHC) Staining

Paraffin‐embedded tissue sections were stained with H&E following standard protocols for histological evaluation. For IHC, sections were deparaffinized, subjected to antigen retrieval and blocking, then incubated at 4°C overnight with primary antibodies: ETV4 (Proteintech, 1:200, Cat. #10684‐1‐AP), ASNS (Proteintech, 1:100, Cat. #14681‐1‐AP), MET (Proteintech, 1:500, Cat. #25869‐1‐AP), and Ki‐67 (Servicebio, 1:200, Cat. #GB111141). After washing with phosphate‐buffered saline with Tween 20 (PBST), HRP‐conjugated secondary antibodies were applied for 1 h at room temperature, followed by DAB (ZSGB‐Bio, Beijing, China, Cat. #ZLI‐9017) color development. Following the chromogenic reaction, slides were counterstained with hematoxylin, dehydrated, and mounted for observation and scanning.

### 4.16 Immunofluorescence (IF)

Immunofluorescence staining was used to assess the subcellular localization and expression of target proteins in cancer cells. Cells were cultured on confocal dishes, fixed with 4% paraformaldehyde for 15 min at room temperature, and permeabilized with 0.5% Triton X‐100 in PBS for 15 min. After blocking with 10% goat serum for 30 min, cells were incubated overnight at 4°C with the indicated primary antibodies, including ETV4 (Proteintech, 1:200, Cat. #10684‐1‐AP), α‐SMA (Proteintech, 1:200, Cat. #67735‐1‐Ig), FAP (Proteintech, 1:200, Cat. #11779‐1‐AP), and IL‐6 (Proteintech, 1:200, Cat. #21865‐1‐AP). For double immunofluorescence staining, primary antibodies from different host species were coincubated. After washing, cells were incubated with FITC‐ and Alexa Fluor 594‐conjugated secondary antibodies for 1 h at room temperature. Nuclei were counterstained with DAPI (Servicebio, Cat. #G1012) for 10 min. Immunofluorescence images were acquired using a fluorescence microscope.

### 4.17 HGF Detection by Enzyme‐Linked Immunosorbent Assay (ELISA)

The concentration of HGF in cell culture supernatants was measured using a Human HGF ELISA Kit (Elabscience, Wuhan, China, Cat. #E‐EL‐H0084) following the manufacturer's protocol. Briefly, samples and standards were added to the precoated plate, followed by sequential incubation with a biotinylated antibody, HRP‐conjugated streptavidin, and tetramethylbenzidine (TMB) substrate. Absorbance was read at 450 nm, and HGF levels were calculated from a standard curve.

### 4.18 Asn Detection

Intracellular and extracellular Asn levels were measured using a commercial colorimetric assay kit (Biosharp, Hefei, China, Cat. #BL1788B) following the manufacturer's protocol. The assay measures the decrease in NADH absorbance at 340 nm, which is proportional to the Asn concentration. For intracellular measurements, cell lysates were prepared. For extracellular measurements, culture medium was collected.

### 4.19 Isolation, Characterization, and Coculture of Primary CRC‐derived CAFs

Fresh CRC tissues were obtained from two patients undergoing surgical resection. Tissues were repeatedly washed with PBS containing 1% penicillin/streptomycin, minced into ∼1 mm^3^ fragments, and enzymatically digested with type IV collagenase (Yeasen, Cat. #40510ES) at 37°C for 3 h with gentle agitation. The resulting cell suspension was filtered through a 70‐µm cell strainer (Yeasen, Cat. #84702ES), centrifuged, and cultured in DMEM/F12 (Servicebio, Cat. #G4610) medium supplemented with 10% FBS. Approximately 2 h after initial plating, the medium was replaced to remove nonadherent cells. When cultures reached ∼80% confluence, cells were briefly digested with 0.25% trypsin; preferentially detached CAFs were immediately neutralized with complete medium and re‐seeded into new culture dishes. This differential adhesion and digestion procedure was repeated to enrich and purify CAFs. The activation status and purity of third‐passage CAFs were validated by immunofluorescence and Western blot analyses. CAFs used in subsequent experiments were required to exhibit the phenotypic characteristics of α‐SMA^+^, FAP^+^, and E‐cadherin^−^. For coculture experiments, CAFs (1 × 10^5^) were seeded into the upper chambers of Transwell inserts with 0.4 µm pore size, while CRC cells (1 × 10^5^) were cultured in the lower chambers.

### 4.20 In Vitro Hepatic Stellate Cell and CAF Polarization

LX‐2 cells (a human HSC line) and primary CRC‐derived CAFs were used to evaluate stromal polarization. Before stimulation, LX‐2 cells were preincubated for 12 h in DMEM supplemented with 2% FBS to induce a quiescent state, while primary CAFs were maintained in DMEM/F12 containing 10% FBS. Cells were then treated for 48 h with various stimuli, including HGF‐treated TCM, l‐Asn (0.1 mM), or recombinant human cytokines (IL‐6, TNF‐α, and IL‐1β; 5 ng/mL each). Total RNA was subsequently extracted to quantify the mRNA expression of iCAF‐associated inflammatory markers (IL1B, IL6, CXCL1, CXCL8, and CCL2) and myCAF‐associated markers (ACTA2, ACTG2, and COL1A1) by RT‐qPCR.

### 4.21 Animal Studies

All animal procedures were approved by the Animal Care and Use Committee of Wuhan University (Approval No. ZN2024199). Male BALB/c nude mice and C57BL/6 mice (4–6 weeks old) were purchased from the Hubei Provincial Center for Experimental Animals (Wuhan, China) and housed in the animal facility of Zhongnan Hospital of Wuhan University under specific pathogen‐free conditions with a 12 h light/dark cycle for 1 week prior to experimentation. For subcutaneous xenograft models, ETV4‐modified human CRC cell lines (HCT116 and DLD‐1; 2 × 10^6^ cells) or ETV4‐knockdown mouse CRC cell line MC38 (2 × 10^5^ cells) were suspended in 0.1 mL PBS and subcutaneously injected into the flanks of BALB/c nude mice or C57BL/6 mice, respectively. For coinjection experiments, HCT116‐shETV4#1 cells (1 × 10^6^) were inoculated either alone or mixed with CAFs (1 × 10^6^) at a 1:1 ratio. Tumor volume was measured every 3–4 days using calipers and calculated using the formula: *V* = length × width^2^ × 0.5. Mice were euthanized approximately 3 weeks post‐injection, and tumors were harvested for IHC. Liver metastasis models were established via intrasplenic injection of the indicated human HCT116 or DLD‐1 cells (1 × 10^6^ cells) into BALB/c nude mice, or MC38 cells (1 × 10^5^ cells) into C57BL/6 mice. Due to varying tumor progression rates, mice were sacrificed at specific endpoints (3 weeks for the MC38 model and 7 weeks for human CRC models), and livers were collected for H&E staining. In accordance with approved protocols, all tumor diameters were maintained below 15 mm.

For therapeutic studies, mice were randomized into treatment groups prior to drug administration. In the subcutaneous model, treatment was initiated when tumor volumes reached 50–100 mm^3^. In the liver metastasis model, drug administration began on day 5 post‐injection. Mice were divided into four groups: (1) Vehicle (PBS); (2) Anti‐MET antibody (Onartuzumab, 30 mg/kg, intraperitoneally every 4 days); (3) ASNase (3 U/g, intraperitoneally every other day); and (4) combination therapy. Tumor size and body weight were monitored throughout the study.

### 4.22 Statistical Analysis

All experiments were performed with at least three independent biological replicates, and data are presented as mean ± standard deviation (SD). Statistical analyses were conducted using GraphPad Prism (v9.0), SPSS (v26.0), and R (v4.2.1). Prior to analysis, normality and homogeneity of variance tests were performed. Two‐tailed unpaired Student's *t*‐tests were used for comparisons between two unpaired groups, while one‐way ANOVA (for a single factor) or two‐way ANOVA (for two independent variables) was employed for multi‐group comparisons. Two‐tailed paired Student's *t*‐tests were used for paired tissue samples. Correlations between gene expression and clinicopathological features were assessed using Chi‐square tests, and Pearson correlation coefficients were calculated to evaluate relationships between two genes or gene signatures. Kaplan–Meier survival analysis was performed, with group comparisons made by the log‐rank test. The sample sizes (*n*) of independent experiments and statistical methods are detailed in the figure legends, and exact *p* values are shown in the figures. *p* < 0.05 was considered statistically significant. All data are available in the article and supplementary materials.

## Author Contributions

D.F., M.Z., M.C., and M.W. contributed equally to this work. Conceptualization and supervision were performed by Y.Q. and Y.W. Methodology was established by D.F. and M.Z. with guidance from Y.Q. and Y.W. The investigation was conducted by D.F., M.Z., M.C., M.W., Z.H., Z.L., Y.L., W.Q., G.C., Y.L., D.W., J.X., and P.Y. Visualization was created by D.F., M.Z., and M.C. The original manuscript was written by D.F., M.Z., M.C., and M.W. Y.Q. and Y.W. reviewed and edited the manuscript. All authors have read and agreed to the published version of the manuscript.

## Conflicts of Interest

The authors declare no conflicts of interest.

## Supporting information




**Supporting File 1**: advs74662‐sup‐0001‐SuppMat.docx.

**Supporting File 2**: advs74662‐sup‐0002‐SuppMat.docx.

## Data Availability

Supporting data for this study are available from the corresponding author upon reasonable request.
