# Supplementary material for: ETV4 Promotes Colorectal Cancer Progression by Reprogramming Asparagine Metabolism to Remodel the Stromal Microenvironment
Source: Adv Sci (Weinh). 2026 Mar 20;13(26):e16557. doi: 10.1002/advs.202516557 (PMC13159137; doi:10.1002/advs.202516557)

**ETV4 Promotes Colorectal Cancer Progression by Reprogramming Asparagine Metabolism to Remodel the Stromal Microenvironment**

**Supporting Information_Original Blots**
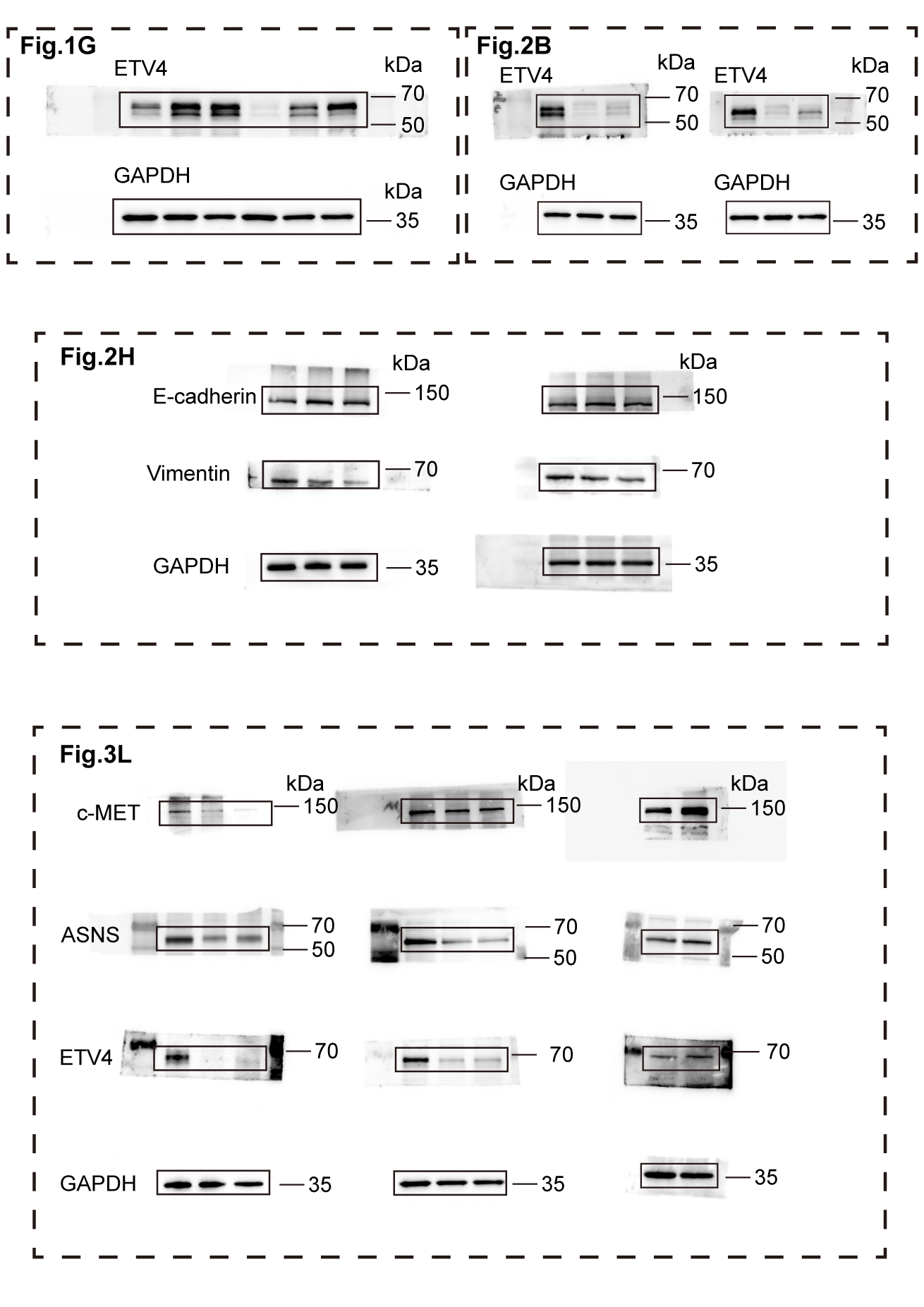

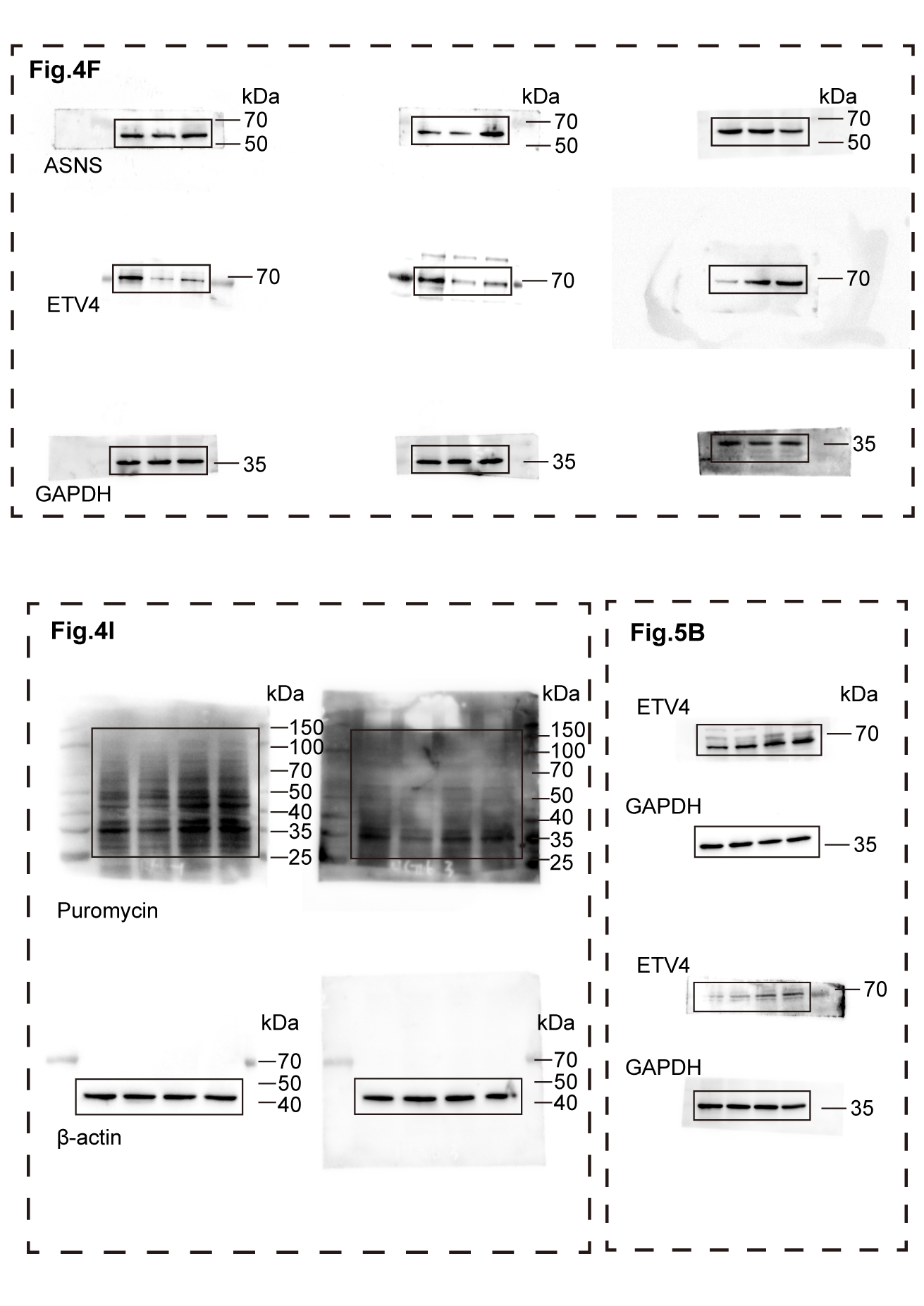

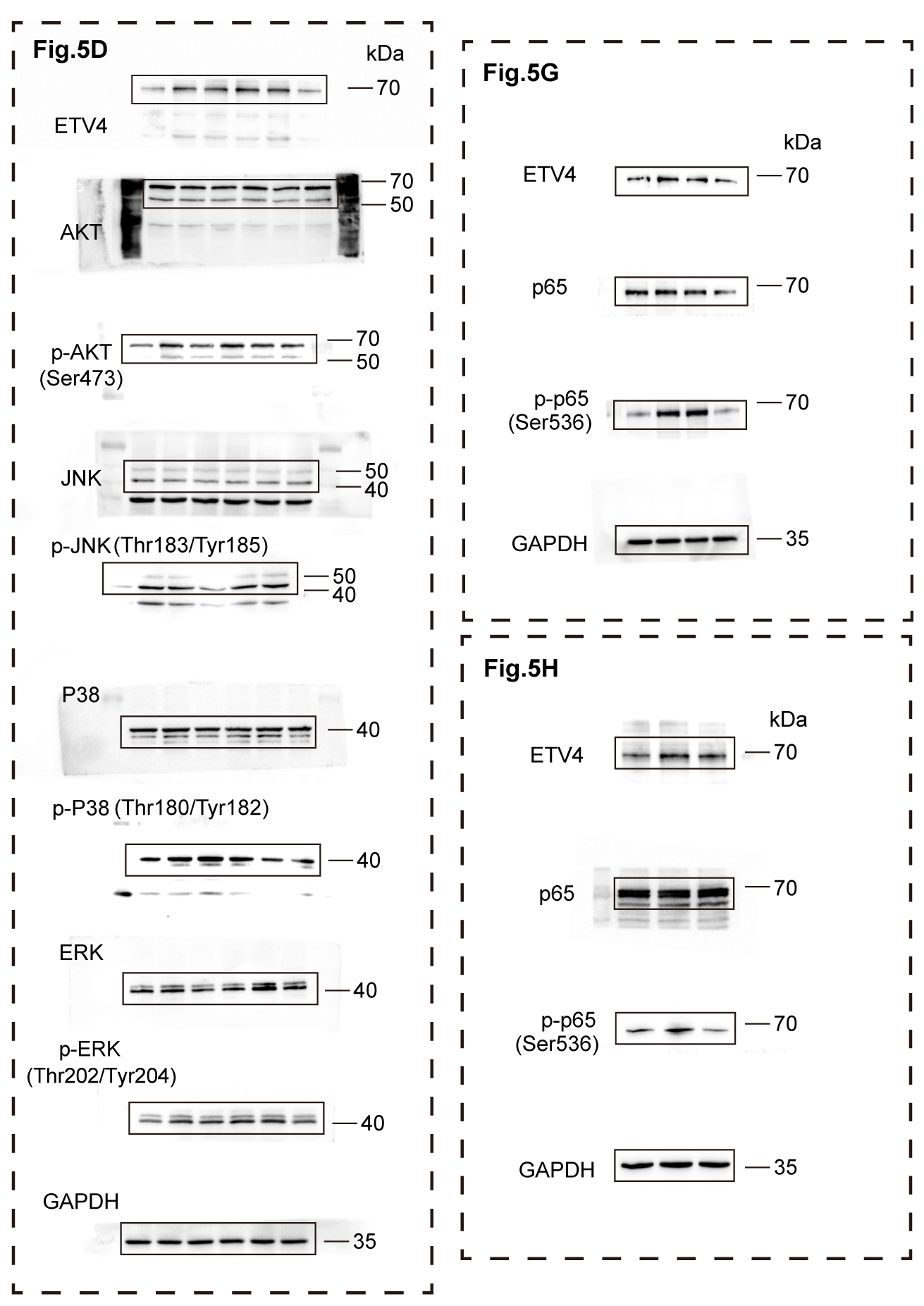

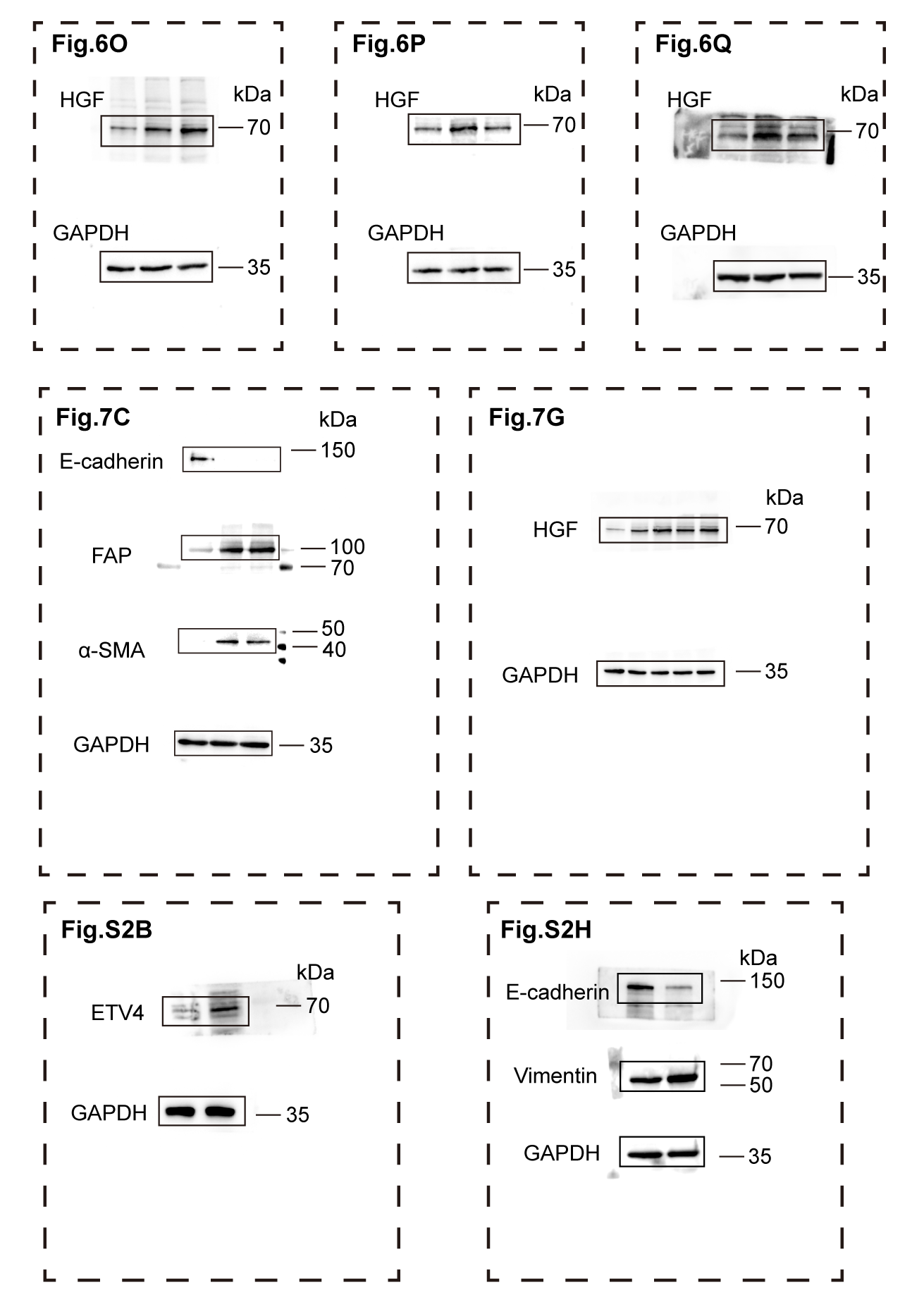

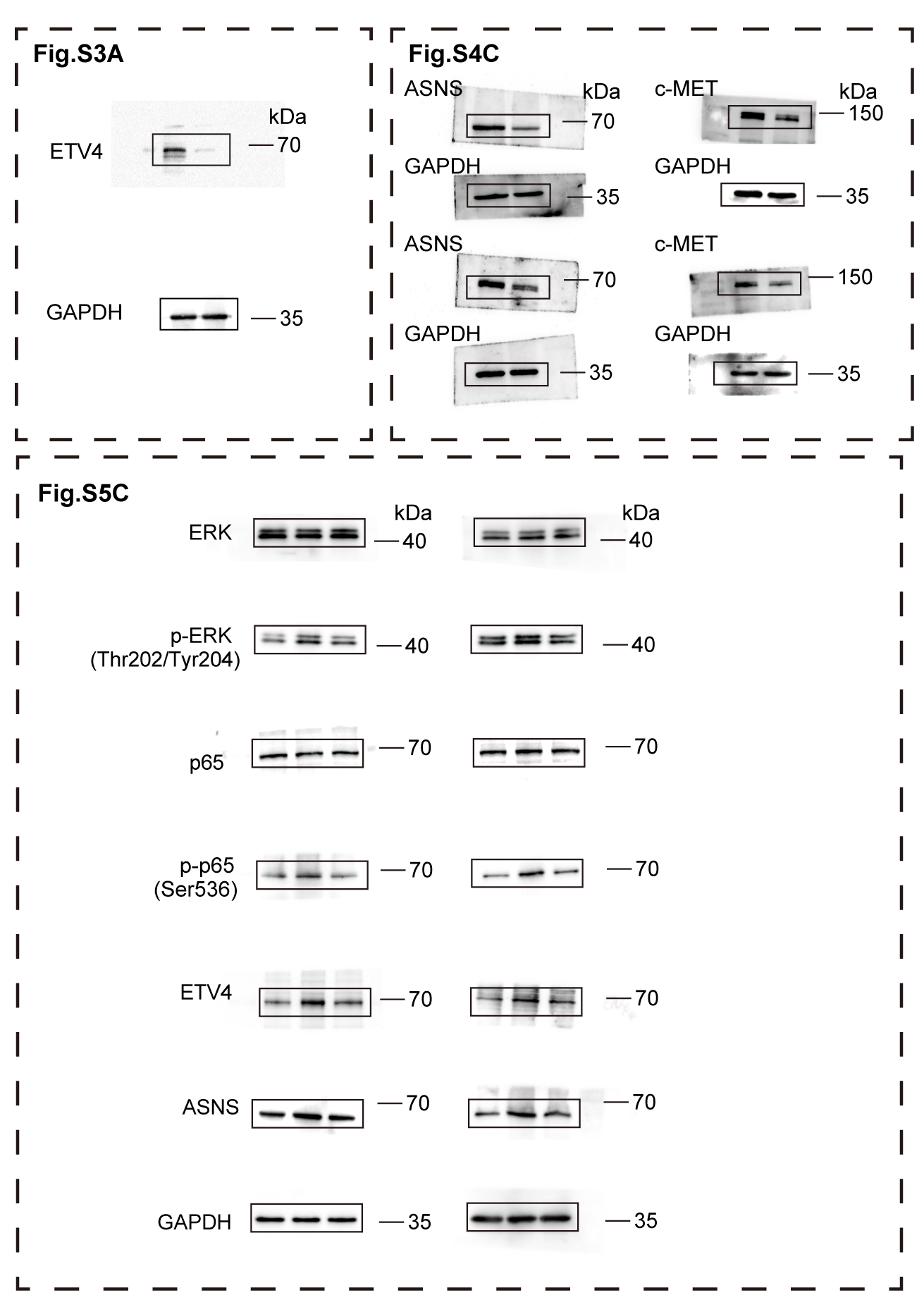

Supplement: Supplementary file 1 — Supporting File 1: advs74662‐sup‐0001‐SuppMat.docx. [file ADVS-13-e16557-s002.docx]
