# Supplementary material for: ETV4 Promotes Colorectal Cancer Progression by Reprogramming Asparagine Metabolism to Remodel the Stromal Microenvironment
Source: Adv Sci (Weinh). 2026 Mar 20;13(26):e16557. doi: 10.1002/advs.202516557 (PMC13159137; doi:10.1002/advs.202516557)
Supplement: Supplementary file 2 — Supporting File 2: advs74662‐sup‐0002‐SuppMat.docx. [file ADVS-13-e16557-s001.docx]

**Supplementary Figure**


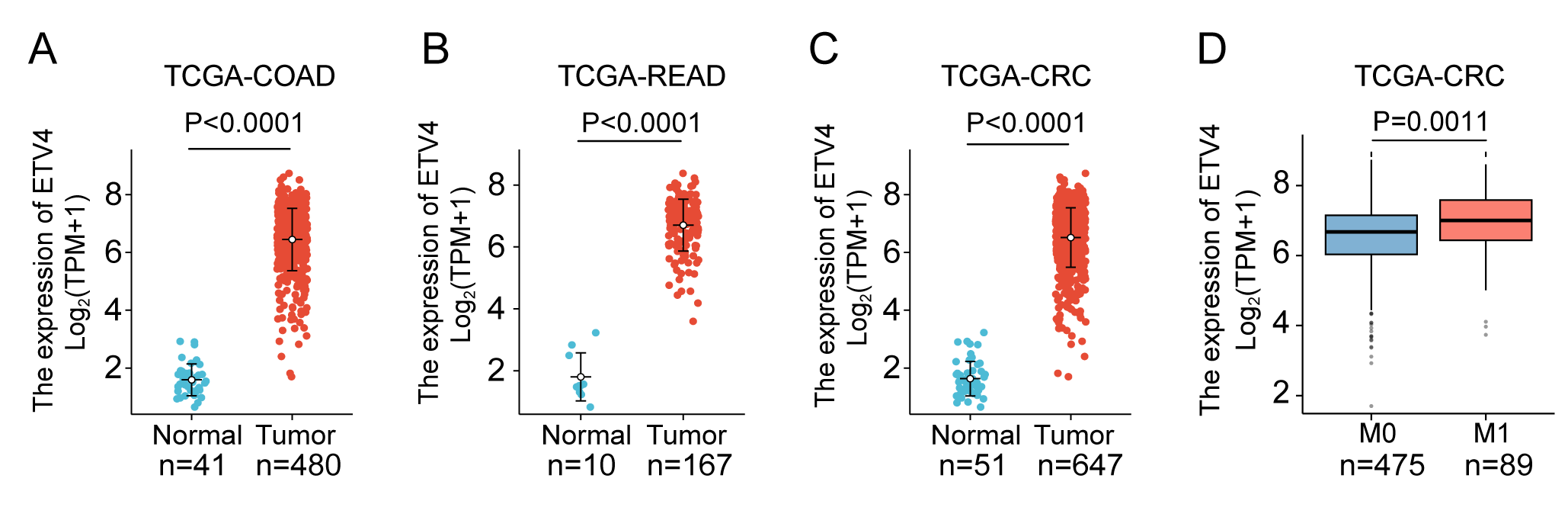


**Figure S1.** ETV4 expression in TCGA colorectal cancer cohorts. A–C) Dot plots showing ETV4 mRNA expression (log2(TPM+1)) in normal and tumor tissues from the TCGA Colon Adenocarcinoma (COAD) cohort (A; normal, n = 41; tumor, n = 480), the TCGA Rectum Adenocarcinoma (READ) cohort (B; normal, n = 10; tumor, n = 167), and the combined TCGA Colorectal Cancer (CRC) cohort (C; normal, n = 51; tumor, n = 647). D) ETV4 mRNA expression in TCGA-CRC tumors stratified by metastatic status: M0 (no distant metastasis, n = 475) and M1 (with distant metastasis, n = 89). Data are presented as mean ± SD (A–D). Statistical significance was assessed by two-tailed unpaired Student’s t-test (A–D). *P* values are indicated in the figure, and *P* < 0.05 was considered statistically significant.


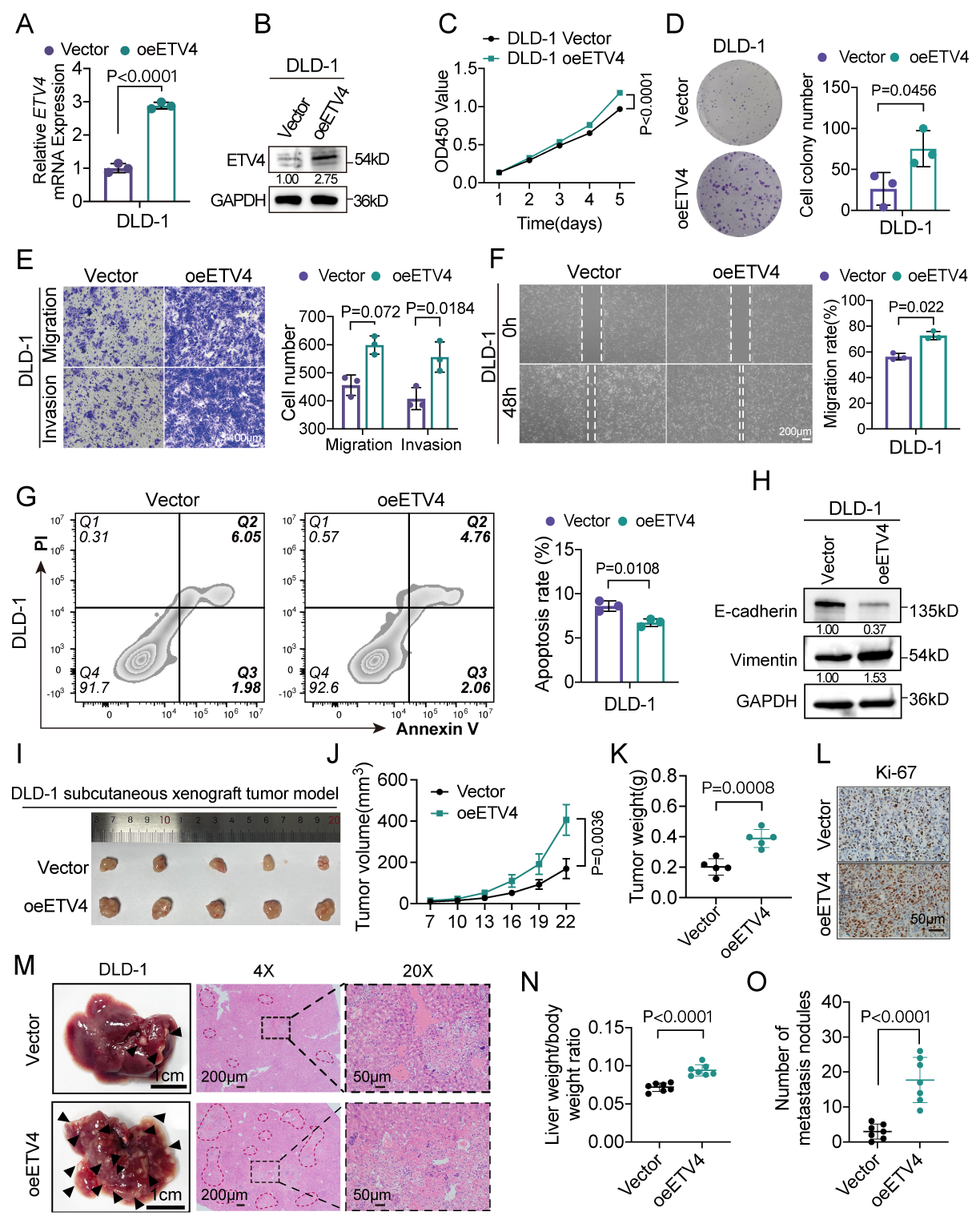


**Figure S2.** ETV4 overexpression accelerates CRC growth and metastasis in vitro and in vivo. A, B) ETV4 overexpression in DLD-1 cells was confirmed by RT-qPCR (A, n = 3) and Western blot (B) following transfection with a control vector or an ETV4-overexpressing construct (oeETV4). C–G) Functional analyses of DLD-1 cells expressing a control vector or oeETV4, including CCK-8 cell proliferation assays (C), colony formation assays (D), Transwell migration and invasion assays (E), wound healing assays (F), and Annexin V-FITC/PI apoptosis assays (G). Quantification is provided for these panels (n = 3). Scale bars, 100 μm (E) and 200 μm (F). H) Western blot analysis of the EMT markers E-cadherin and Vimentin. I–L) In vivo assays using subcutaneous xenograft models: images of tumors from vector and oeETV4 groups (I), tumor growth curves (J) and tumor weight (K) at the endpoint (n = 5 mice per group), and Ki-67 IHC of xenograft tumors (L, scale bar, 50 μm). M–O) Liver metastasis model: representative gross liver images and H&E-stained sections at low magnification (4×, scale bar, 200 μm) and high magnification (20×, scale bar, 50 μm) (M), with quantification of liver-to-body weight ratio (N) and the number of metastatic nodules (O) (n = 7 mice per group). Data are presented as mean ± SD (A, C–G, J, K, N, O), and statistical significance was determined by two-tailed unpaired Student’s t-test (A, D–G, K, N, O) or two-way ANOVA with Sidak’s multiple comparisons test (C, J). *P* values are provided in the figure, and *P* < 0.05 was considered statistically significant.


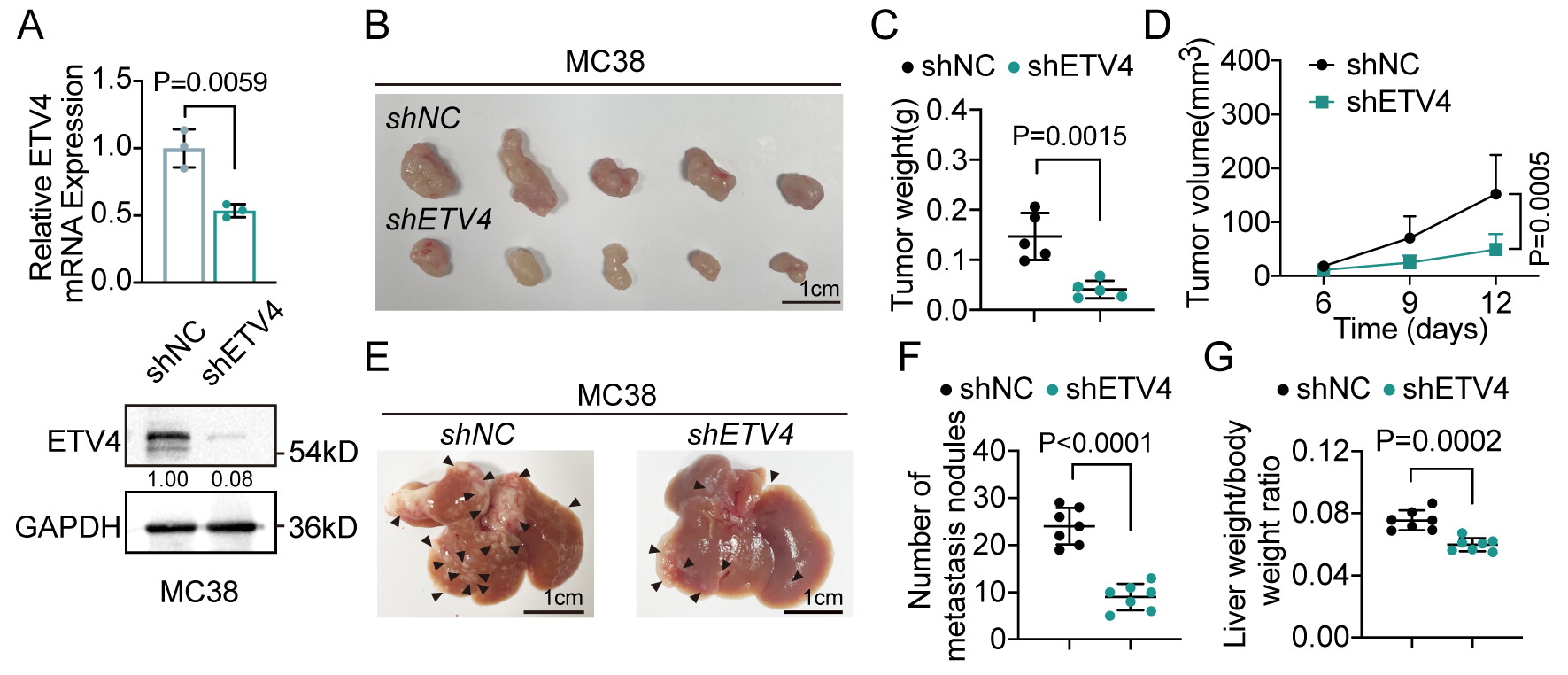


**Figure S3.** ETV4 silencing suppresses tumor growth and liver metastasis in an immunocompetent syngeneic mouse model. A) ETV4 knockdown efficiency in MC38 cells was validated by RT-qPCR (n = 3) and Western blot. B–D) Subcutaneous tumor model. MC38 cells stably expressing shNC or shETV4 were subcutaneously injected into C57BL/6 mice (n = 5 mice per group). Shown are images of tumors (B), tumor weight (C), and tumor growth curves (D). E–G) Liver metastasis model. MC38-shNC or shETV4 cells were injected into the spleen of C57BL/6 mice to establish liver metastases (n = 7 mice per group). Shown are representative gross images of livers (E, scale bar, 1 cm), quantification of liver metastatic nodules (F), and liver-to-body weight ratio (G). Data are presented as mean ± SD (A, C, D, F, G). Statistical significance was determined by unpaired Student’s t-test (A, C, F, G) or two-way ANOVA with Sidak’s multiple comparisons test (D). *P* values are indicated in the figure, and *P* < 0.05 was considered statistically significant.


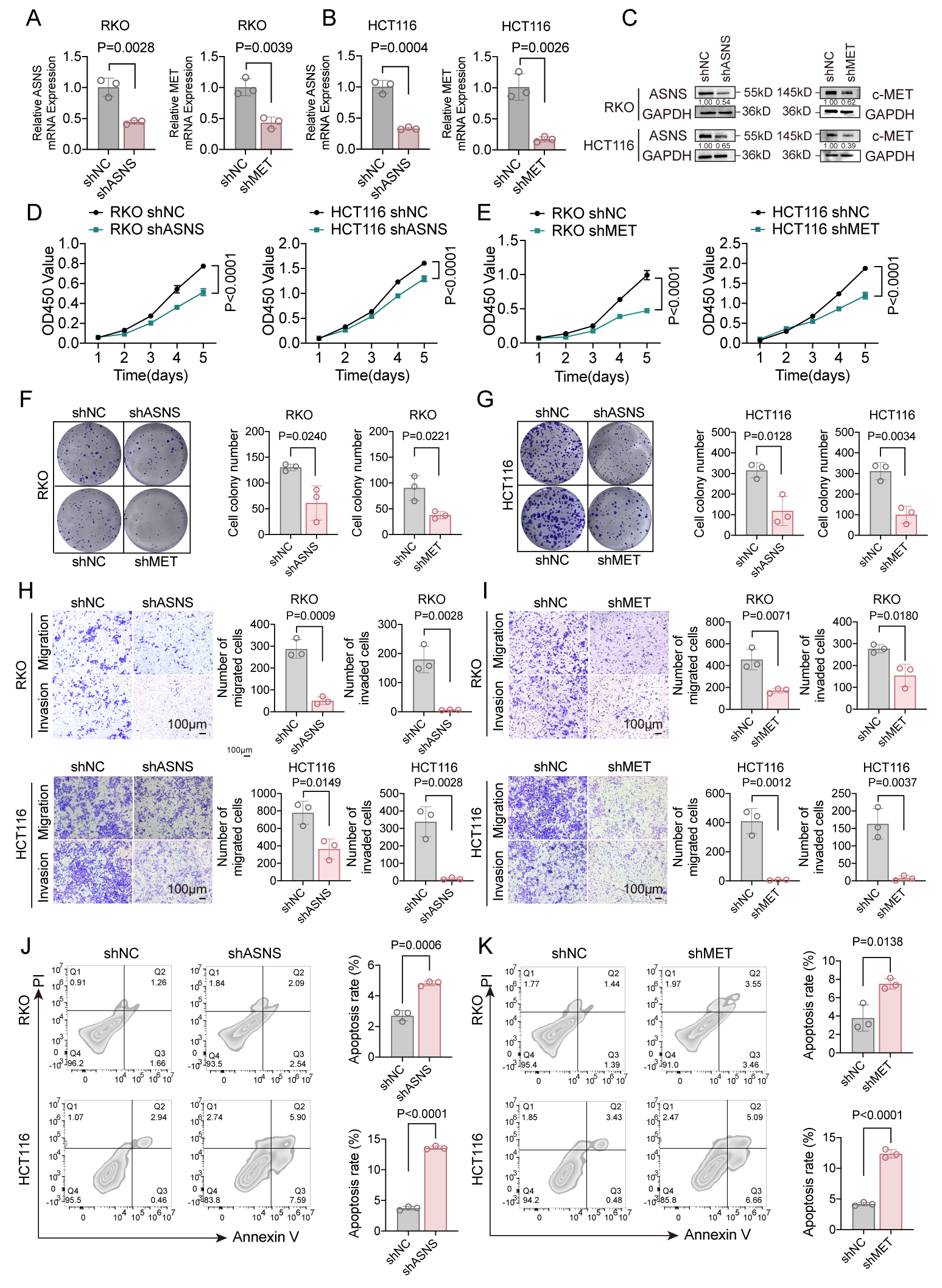


**Figure S4.** Silencing of ASNS or MET phenocopies ETV4 knockdown in CRC cells. A, B) RT-qPCR analysis of ASNS and MET mRNA levels in RKO (A) and HCT116 (B) cells transfected with control shRNA (shNC), shASNS, or shMET (n = 3). C) Western blot analysis of ASNS and MET protein levels following their respective silencing in RKO and HCT116 cells. D–K) Silencing of ASNS or MET suppressed malignant phenotypes in CRC cells. This was evidenced by impaired cell proliferation (CCK-8 assay; D, E), reduced colony formation (F, G), diminished migration and invasion (Transwell assays; H, I; scale bar, 100 μm), and increased apoptosis (flow cytometry; J, K) in both RKO and HCT116 cells. Quantification is shown to the right of representative images (n = 3). Data are presented as mean ± SD (A, B, D–K). Statistical significance was determined by two-tailed unpaired Student’s t-test (A, B, F–K) or two-way ANOVA followed by Sidak’s multiple comparisons test (D, E). *P* values are indicated in the figure, and *P* < 0.05 was considered statistically significant.


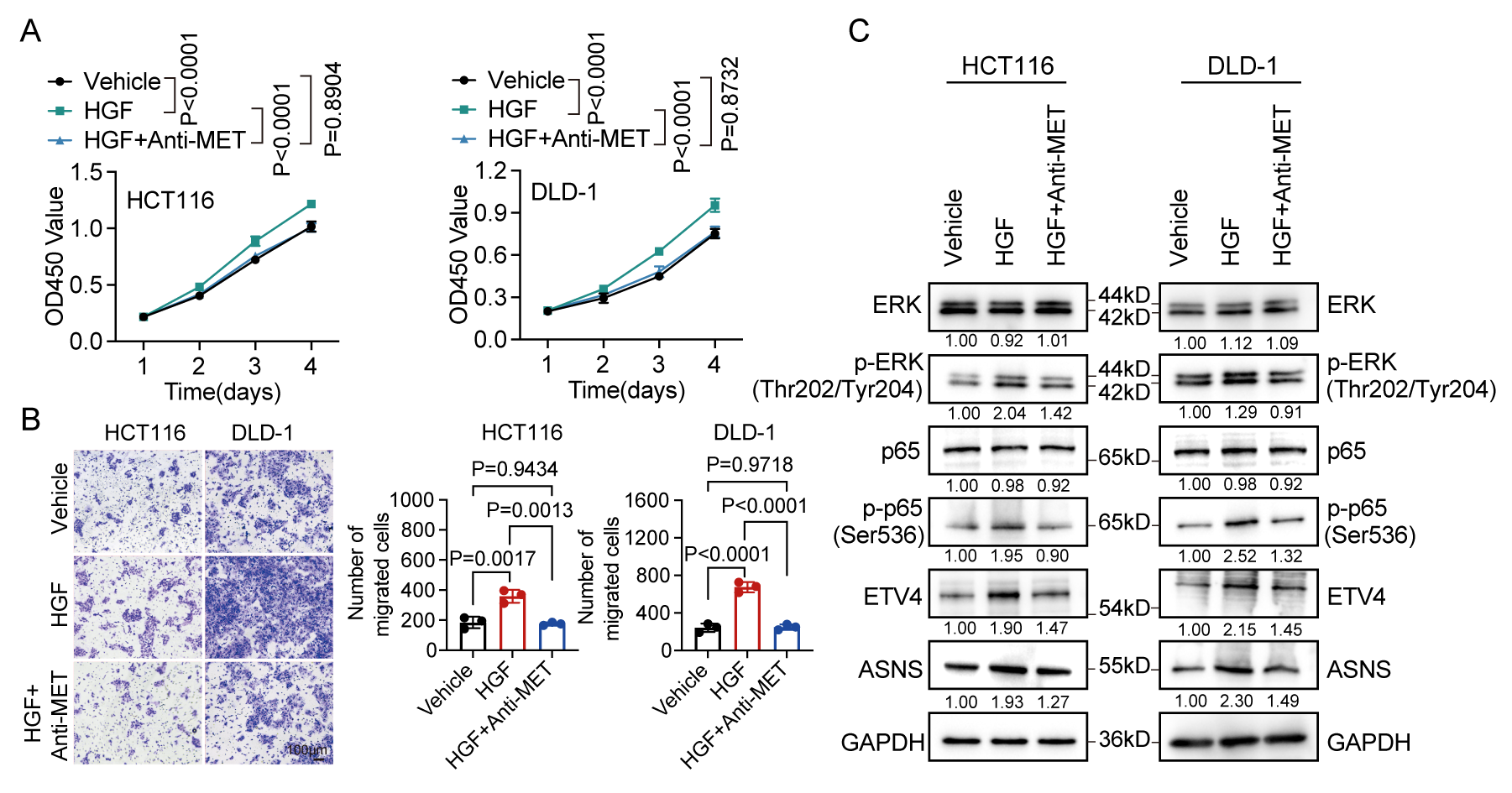


**Figure S5.** Onartuzumab specifically blocks HGF/MET-driven cell proliferation, migration, and downstream ERK1/2–p65–ETV4 signaling. A) The proliferation of HCT116 and DLD-1 cells was measured by a CCK-8 assay. Cells were either left untreated (Control), stimulated with HGF (40 ng/mL), or pre-treated with the MET antibody Onartuzumab (10 µg/mL) for 2 h prior to HGF stimulation (n = 3). B) Transwell migration of HCT116 and DLD-1 cells under the treatment groups in (A). Representative images and quantified results are provided (n = 3). Scale bar, 100 μm. C) Western blot analysis of total and phosphorylated ERK1/2, total and phosphorylated p65, ETV4, ASNS, and GAPDH in HCT116 and DLD-1 cells under the treatment groups in (A). Data are presented as mean ± SD (A, B). Statistical significance was determined by two-way ANOVA with Tukey’s multiple comparisons test (A), or one-way ANOVA with Tukey’s multiple comparisons test (B). *P* values are provided in the figure, and *P* < 0.05 was considered statistically significant.


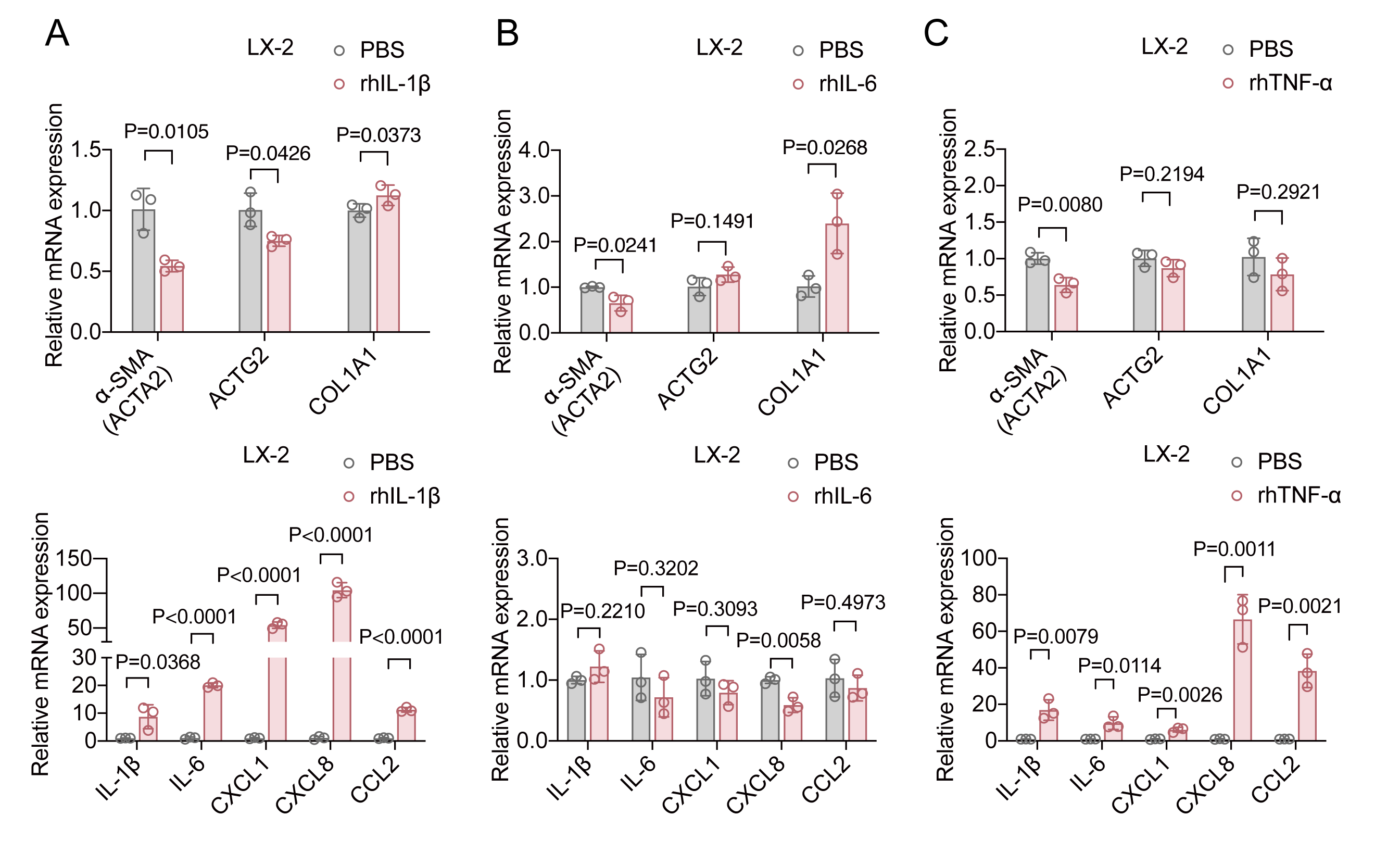


**Figure S6.** Differential regulation of the hepatic stellate cells (HSCs) phenotype by inflammatory cytokines. A–C) LX-2 HSCs were treated for 48 h with 5 ng/mL of rhIL-1β (A), rhIL-6 (B), or rhTNF-α (C), and the expression of myCAF-associated markers (top) and iCAF-associated inflammatory markers (bottom) was assessed by RT-qPCR (n = 3). Data are presented as mean ± SD (A–C). Statistical significance was determined by a two-tailed unpaired Student’s t-test (A–C). *P* values are indicated in the figure, and *P* < 0.05 was considered statistically significant.


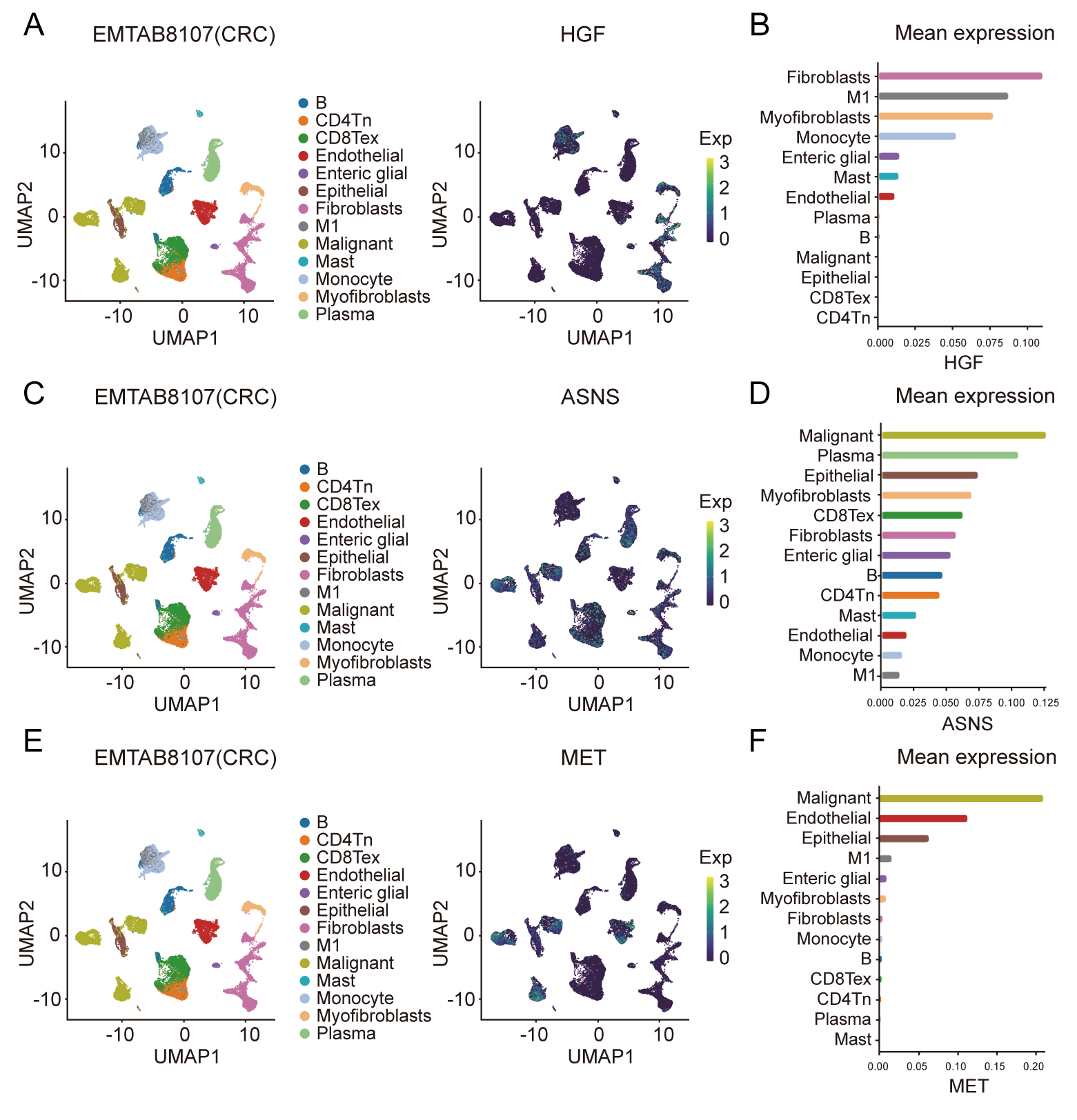


**Figure S7.** Single-cell transcriptomic distribution of HGF, ASNS, and MET in the CRC microenvironment. A, C, E) UMAP plots from scRNA-seq data (EMTAB8107) showing the expression of HGF (A), ASNS (C), and MET (E) across annotated cell populations. B, D, F) Bar plots showing the average expression levels of HGF (B), ASNS (D), and MET (F) in different cell types, highlighting that ASNS and MET show the highest expression in malignant epithelial cells, while HGF is most highly expressed in fibroblasts.


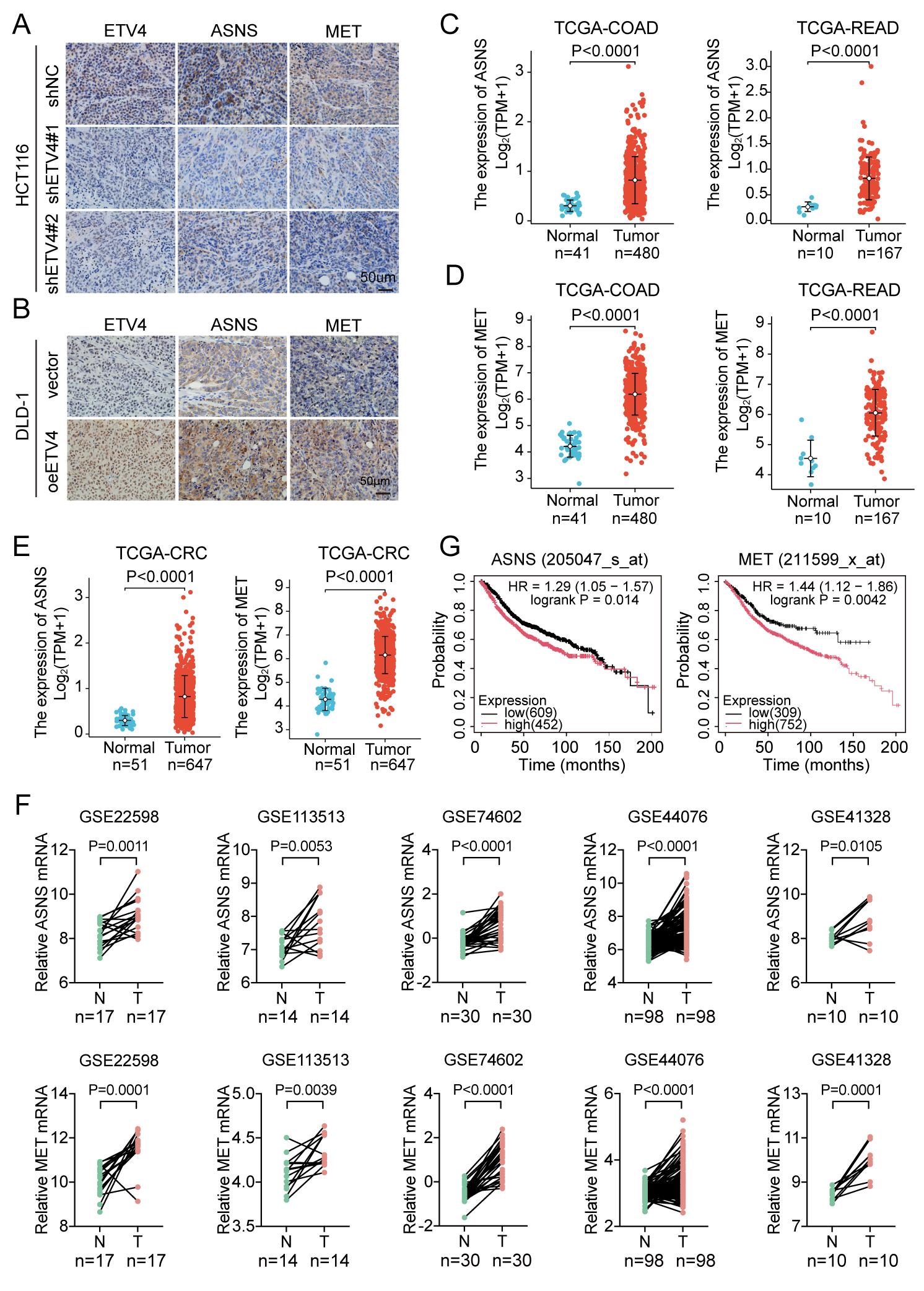


**Figure S8.** Validation of ETV4 regulation of ASNS and MET expression and their expression patterns and prognostic value in CRC. A, B) IHC analysis of ETV4, ASNS, and MET protein expression in xenograft tumors derived from HCT116-shETV4 cells (A) and DLD-1-oeETV4 cells (B). Scale bar, 50 μm. C, D) ASNS and MET transcript levels in tumor versus normal tissues from TCGA-COAD (C) and TCGA-READ (D) cohorts. E) Expression levels of ASNS and MET in TCGA-CRC tumors and normal tissues. F) ASNS and MET mRNA levels in paired CRC tumor and normal tissues across multiple GEO datasets. G) Kaplan–Meier survival analysis showing the association of ASNS and MET expression with overall survival in CRC patients. Data are presented as mean ± SD (C–E). Sample sizes (n) are provided in the figure (C–G). Statistical significance was assessed by two-tailed unpaired Student’s t-test (C–E), two-tailed paired Student’s t-test (F), or log-rank test (G). *P* values are indicated in the figure, and *P* < 0.05 was considered statistically significant.


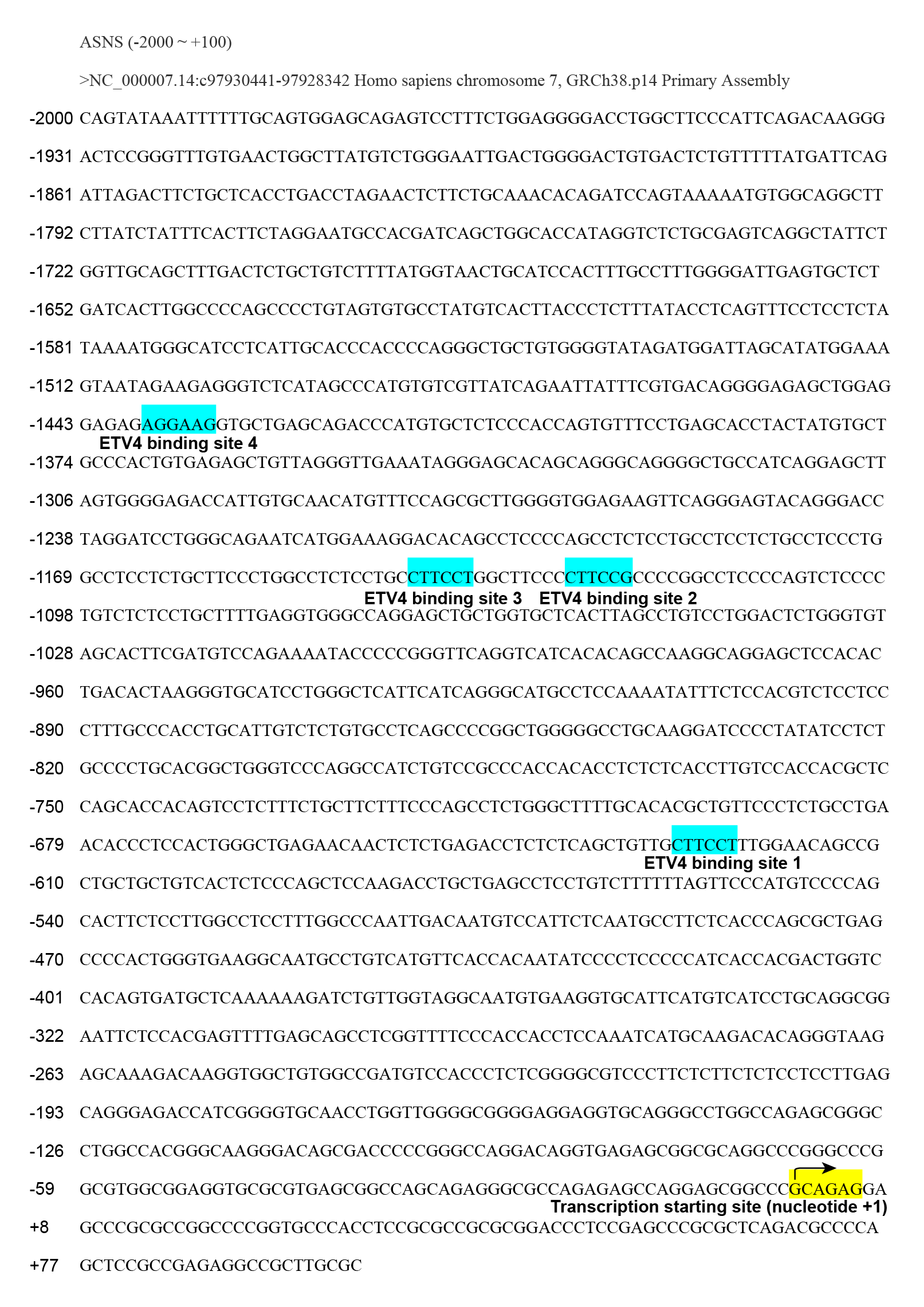


**Figure S9.** Sequence of the human ASNS promoter, with putative ETV4 binding sites highlighted in blue and the transcription start site (TSS) indicated in yellow.


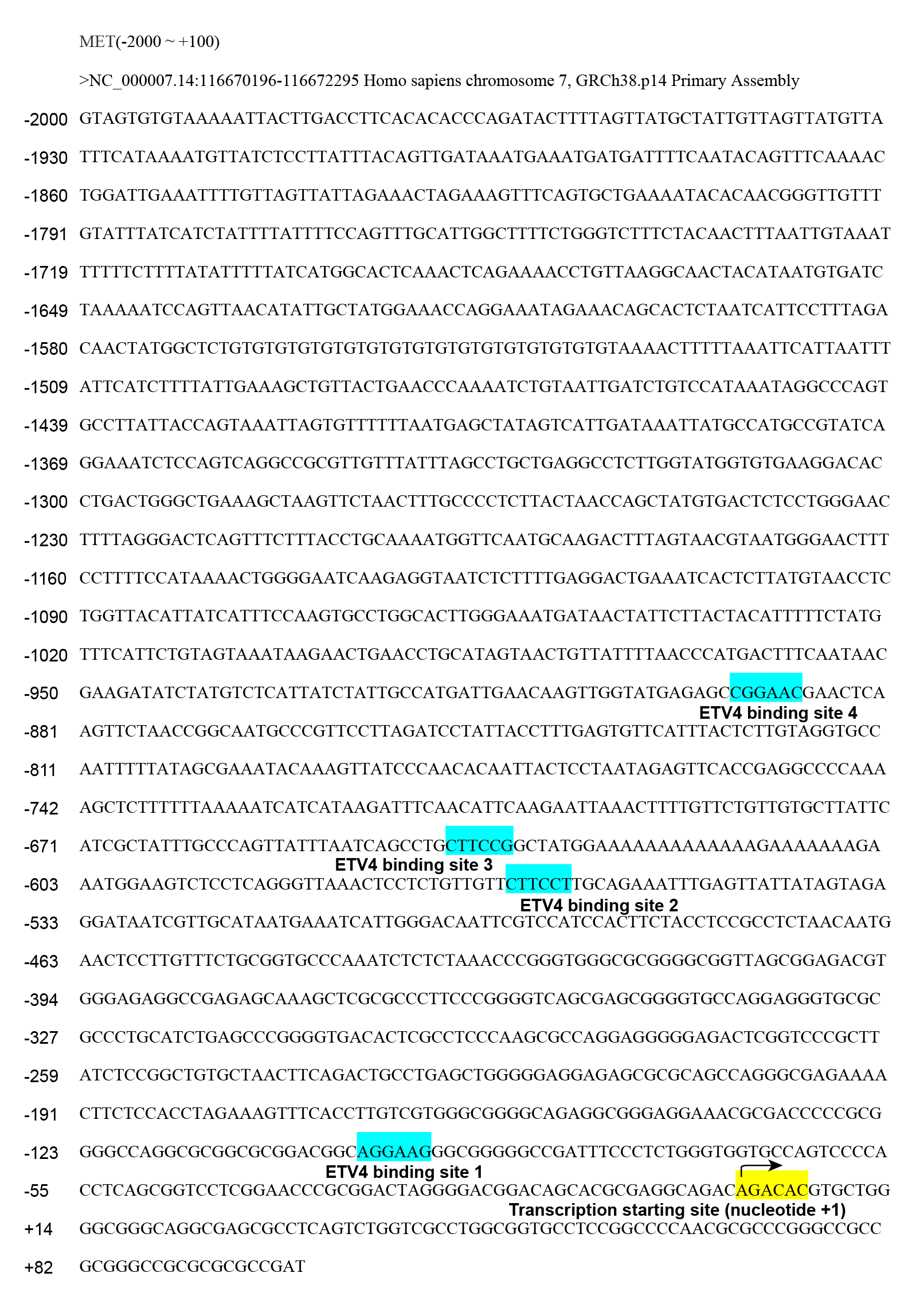


**Figure S10.** Sequence of the human MET promoter, with putative ETV4 binding sites highlighted in blue and the transcription start site (TSS) indicated in yellow.


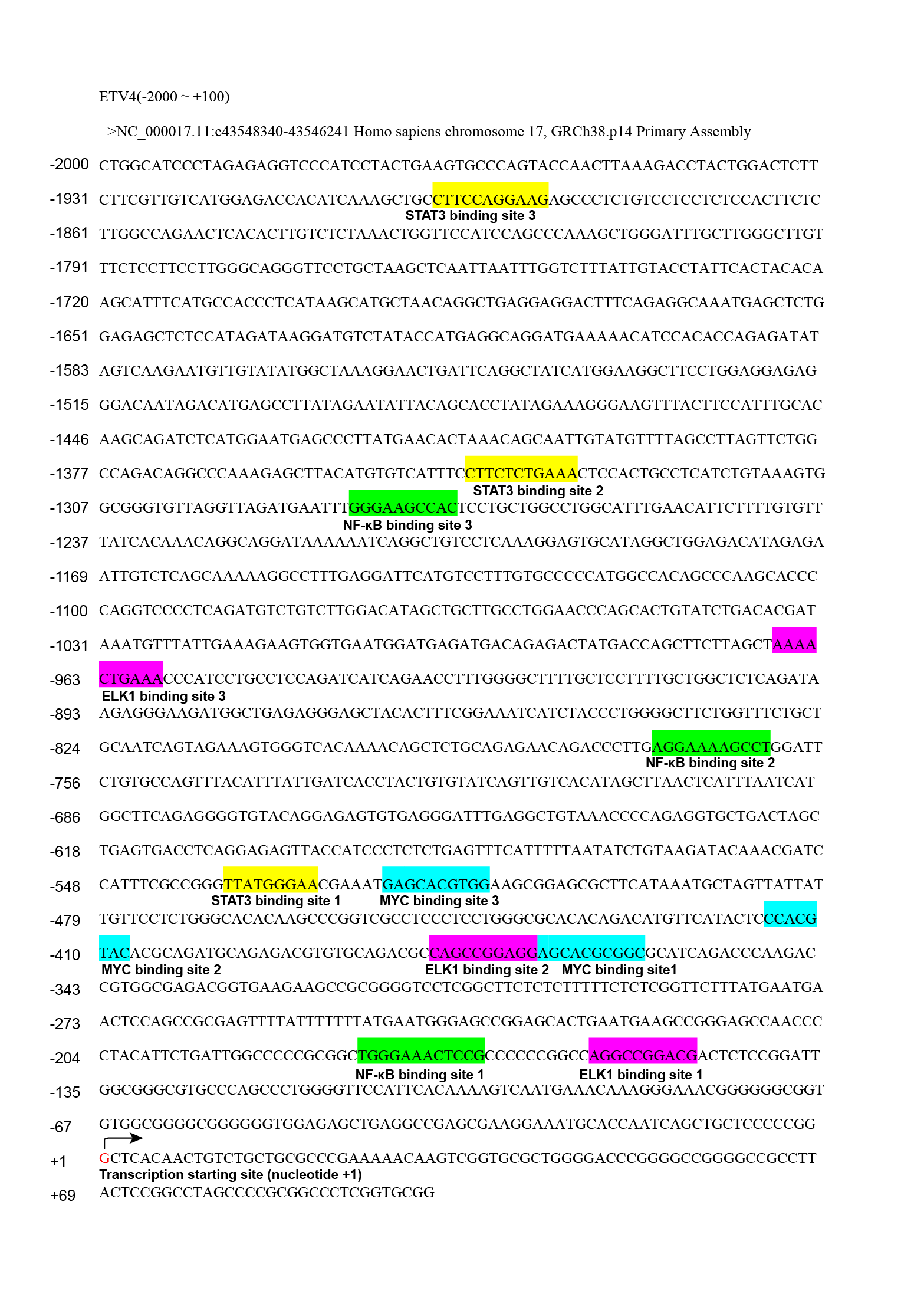


**Figure S11.** Sequence of the human ETV4 promoter, with predicted binding sites for STAT3 (yellow), MYC (blue), ELK1 (purple), and NF-κB (RelA/p65, green) highlighted, along with the transcription start site (TSS) in red.

**Supplementary Table**

**Table S1**. Target sequences of shRNAs used in this study.

| Target gene | shRNA | Species | Target sequence (5' to 3') |
| --- | --- | --- | --- |
| ETV4 | shETV4#1 | Human | CCCTGTGTACATATAAATGAA |
|  | shETV4#2 | Human | CCAGGATCTAAGTCACTTCCA |
|  | shETV4#3 | Human | GCTCCGATACTATTATGAGAA |
| ASNS | shASNS | Human | CGAGTGAAGAAATATCCGTAT |
| MET | shMET | Human | TCAACTTCTTTGTAGGCAATA |
| MYC | shMYC | Human | CCTGAGACAGATCAGCAACAA |
| ELK1 | shELK1 | Human | CCTGCTTCCTACGCATACATT |
| RELA | shRELA | Human | CACCATCAACTATGATGAGTT |
| STAT3 | shSTAT3 | Human | GCACAATCTACGAAGAATCAA |
| ETV4 | shETV4 | Mouse | TCGGCCACAGAGGTGGATATT |

**Table S2.** Primer sequences used for RT-qPCR.

| Gene | Species | Sense sequence (5' to 3') | Antisense sequence (5' to 3') |
| --- | --- | --- | --- |
| ETV4 | Human | CAGTGCCTTTACTCCAGTGCC | CTCAGGAAATTCCGTTGCTCT |
|  | Mouse | CATTCCCAGATGATGTCTGCAT | CCACAGTTGTAAGGCACCCC |
| ASNS | Human | GGAAGACAGCCCCGATTTACT | AGCACGAACTGTTGTAATGTCA |
| MET | Human | AGCAATGGGGAGTGTAAAGAGG | CCCAGTCTTGTACTCAGCAAC |
| GAPDH | Human | CCAGGTGGTCTCCTCTGACTTC | GTGGTCGTTGAGGGCAATG |
|  | Mouse | AGGTCGGTGTGAACGGATTTG | GGGGTCGTTGATGGCAACA |
| SLC39A10 | Human | TTTCACTCACATAACCACCAGC | GTGATGACGTAGGCGGTGATT |
| ASNSD1 | Human | AGTGGTAGCAAATGAAGCCAAA | ACTGCAATGAGTCTCCAATGC |
| EIF5A2 | Human | GGACGACCATGCAAAATAGTGG | TGCCCGTGAAAATATCAATTCCA |
| DDIT4 | Human | TGAGGATGAACACTTGTGTGC | CCAATCTGGCTAGGCATCAGC |
| ENC1 | Human | GCTGCTGTCTGATGCACAC | AGAGTTGCACTACCATGTCCT |
| GGCT | Human | GGCATGGAGGGATAGCCAC | CCCCTTCTTGCTCATCCAGAG |
| ELK1 | Human | TCCCTGCTTCCTACGCATACA | GCTGCCACTGGATGGAAACT |
| MYC | Human | GGCTCCTGGCAAAAGGTCA | CTGCGTAGTTGTGCTGATGT |
| RELA | Human | GTGGGGACTACGACCTGAATG | GGGGCACGATTGTCAAAGATG |
| STAT3 | Human | ACCAGCAGTATAGCCGCTTC | GCCACAATCCGGGCAATCT |
| ACTA2 | Human | CTATGAGGGCTATGCCTTGCC | GCTCAGCAGTAGTAACGAAGGA |
| ACTG2 | Human | ATTGTGCGAGACATCAAGGAG | CCATGCCAATAAAGGAAGGCT |
| COL1A1 | Human | GAGGGCCAAGACGAAGACATC | CAGATCACGTCATCGCACAAC |
| IL6 | Human | ACTCACCTCTTCAGAACGAATTG | CCATCTTTGGAAGGTTCAGGTTG |
| IL1B | Human | CCACAGACCTTCCAGGAGAATG | GTGCAGTTCAGTGATCGTACAGG |
| CXCL1 | Human | AGCTTGCCTCAATCCTGCATCC | TCCTTCAGGAACAGCCACCAGT |
| CXCL8 | Human | ACTGAGAGTGATTGAGAGTGGAC | AACCCTCTGCACCCAGTTTTC |
| CCL2 | Human | CAGCCAGATGCAATCAATGCC | TGGAATCCTGAACCCACTTCT |

**Table S3.** Antibodies used for Western blot analysis.

| Antibody | Host | Supplier | Catalog No. | Dilution |
| --- | --- | --- | --- | --- |
| ETV4 | Rabbit | Proteintech | 10684-1-AP | 1:1000 |
| ASNS | Rabbit | Proteintech | 14681-1-AP | 1:1000 |
| MET | Rabbit | Proteintech | 25869-1-AP | 1:500 |
| HGF | Rabbit | Proteintech | 26881-1-AP | 1:500 |
| E-Cadherin | Rabbit | Cell Signaling Technology | 3195 | 1:1000 |
| Vimentin | Rabbit | Cell Signaling Technology | 5741 | 1:1000 |
| AKT | Rabbit | Proteintech | 10176-2-Ig | 1:5000 |
| p-AKT (Ser473) | Mouse | Proteintech | 66444-1-Ig | 1:2000 |
| JNK | Rabbit | Proteintech | 51151-1-AP | 1:1000 |
| p-JNK (Thr183/Tyr185) | Mouse | Proteintech | 60666-1-Ig | 1:5000 |
| p38 | Rabbit | Proteintech | 14064-1-AP | 1:2000 |
| p-p38 (Thr180/Tyr182) | Rabbit | Proteintech | 28796-1-AP | 1:1000 |
| ERK1/2 | Rabbit | Proteintech | 11257-1-AP | 1:2000 |
| p-ERK1/2 (Thr202/Tyr204) | Rabbit | Proteintech | 28733-1-AP | 1:1000 |
| p65 (NF-κB) | Rabbit | Proteintech | 10745-1-AP | 1:1000 |
| p-p65 (Ser536) | Rabbit | Proteintech | 80379-2-RR | 1:2000 |
| Puromycin | Rabbit | ABclonal | A21205 | 1:2000 |
| α-SMA | Mouse | Proteintech | 67735-1-Ig | 1:20000 |
| FAP | Rabbit | Proteintech | 11779-1-AP | 1:1000 |
| GAPDH | Mouse | Proteintech | 60004-1-Ig | 1:10000 |
| β-Actin | Mouse | Proteintech | 66009-1-Ig | 1:10000 |

**Table S4.** Primer sequences for dual-luciferase reporter assay constructs.

| Primer name | Sequence (5' to 3') | Enzyme |
| --- | --- | --- |
| Primers for ASNS promoter construct | |  |
| (-2000/+100) ASNS sense | GATAGGTACCCAGTATAAATTTTTTGCA | KpnI |
| (-1223/+100) ASNS sense | GATAGGTACCATCATGGAAAGGACACAG | KpnI |
| (-736/+100) ASNS sense | GATAGGTACCTCTTTCTGCTTCTTTCCC | KpnI |
| (-282/+100) ASNS sense | GATAGGTACCATGCAAGACACAGGGTAA | KpnI |
| Antisense | AGATCTCGAGGCGCAAGCGGCCTCTCGG | XhoI |
| Primers for site-directed mutagenesis of the ASNS promoter targeting the ETV4 binding site | |  |
| Binding site 4 mutation sense | AGGAGAGAatgcGGTGCTGAGCAGACCCATG |  |
| Binding site 4 mutation antisense | TCAGCACCgcatTCTCTCCTCCAGCTCTCCCCT |  |
| Binding site 3 mutation sense | CTCCTGCCgcatTGGCTTCCCCTTCCGCCCCG |  |
| Binding site 3 mutation antisense | GGAAGCCAatgcGGCAGGAGAGGCCAGGGAA |  |
| Binding site 2 mutation sense | GCTTCCCCgcatGCCCCGGCCTCCCCAGTCTC |  |
| Binding site 2 mutation antisense | GAGGCCGGGGCatgcGGGGAAGCCAGGAAGGCAGGAGA |  |
| Binding site 1 mutation sense | GCTGTTGCgcatTTTGGAACAGCCGCTGCTG |  |
| Binding site 1 mutation antisense | GTTCCAAAatgcGCAACAGCTGAGAGAGGTC |  |
| Primers for MET promoter construct | |  |
| (-2000/+100) MET sense | GATAGGTACCTAGTGTGTAAAAATTACT | KpnI |
| (-773/+100) MET sense | GATAGGTACCACTCCTAATAGAGTTCAC | KpnI |
| (-703/+100) MET sense | GATAGGTACCAATGGAAGTCTCCTCAGG | KpnI |
| (-247/+100) MET sense | GATAGGTACCGCTAACTTCAGACTGCCT | KpnI |
| (-8/+100) MET sense | GATAGGTACCAGGCAGACAGACACGTGC | KpnI |
| Antisense | AGATCTCGAGATCGGCGCGCGCGGCCCG | XhoI |
| Primers for site-directed mutagenesis of the MET promoter targeting the ETV4 binding site | |  |
| Binding site 4 mutation sense | TGAGAGCCatgcCGAACTCAAGTTCTAACCGG |  |
| Binding site 4 mutation antisense | TGAGTTCGgcatGGCTCTCATACCAACTTGTT |  |
| Binding site 3 mutation sense | GCCTGCgcatGGCTATGGAAAAAAAAAAAAAG |  |
| Binding site 3 mutation antisense | CCATAGCCatgcGCAGGCTGATTAAATAACTG |  |
| Binding site 2 mutation sense | TGTTGTTCgcatTTGCAGAAATTTGAGTTATT |  |
| Binding site 2 mutation antisense | TTCTGCAAatgcGAACAACAGAGGAGTTTAAC |  |
| Binding site 1 mutation sense | GACGGCAatgcGGGCGGGGGCCGATTTCCCTC |  |
| Binding site 1 mutation antisense | CCCCGCCCgcatTGCCGTCCGCGCCG |  |
| Primers for ETV4 promoter construct | |  |
| (-2000/+100) ETV4 sense | GATAGGTACCCTGGCATCCCTAGAGAGG | KpnI |
| Antisense | AGATCTCGAGCCGCACCGAGGGCCGCGG | XhoI |

**Table S5.** Primer sequences used for ChIP-qPCR.

| Promoter | Target Region | Primer | Sequence (5' to 3') |
| --- | --- | --- | --- |
| ASNS | ETV4 Binding Site 3 | ASNS-ChIP-F | GCCTCTCCTGCCTCCTCTGC |
|  |  | ASNS-ChIP-R | GCAGCTCCTGGCCCACCT |
| MET | ETV4 Binding Site 1 | MET-ChIP-F | AGTTTCACCTTGTCGTGGG |
|  |  | MET-ChIP-R | TAGTCCGCGGGTTCCGA |

**Table S6.** Relationship between ETV4 expression and clinical pathological characteristics of patients in the TCGA-CRC cohort.

| Characteristics | Low expression of ETV4 (n = 322) | High expression of ETV4 (n = 322) | P-value (χ²) |
| --- | --- | --- | --- |
| Age, n (%) |  |  | 0.873 |
| ≤ 65 | 139 (21.6%) | 137 (21.3%) |  |
| > 65 | 183 (28.4%) | 185 (28.7%) |  |
| Gender, n (%) |  |  | 0.236 |
| Female | 158 (24.5%) | 143 (22.2%) |  |
| Male | 164 (25.5%) | 179 (27.8%) |  |
| Pathologic T stage, n (%) |  |  | 0.784 |
| T1&T2 | 64 (10%) | 67 (10.5%) |  |
| T3&T4 | 256 (39.9%) | 254 (39.6%) |  |
| Pathologic N stage, n (%) |  |  | 0.021 |
| N0 | 199 (31.1%) | 169 (26.4%) |  |
| N1&N2 | 122 (19.1%) | 150 (23.4%) |  |
| Pathologic M stage, n (%) |  |  | 0.034 |
| M0 | 245 (43.4%) | 230 (40.8%) |  |
| M1 | 35 (6.2%) | 54 (9.6%) |  |

**Table S7.** Relationship between ASNS expression and clinical pathological characteristics of patients in the TCGA-CRC cohort.

| Characteristics | Low expression of ASNS (n = 322) | High expression of ASNS (n = 322) | P-value (χ²) |
| --- | --- | --- | --- |
| Age, n (%) |  |  | 0.873 |
| ≤ 65 | 137 (21.3%) | 139 (21.6%) |  |
| > 65 | 185 (28.7%) | 183 (28.4%) |  |
| Gender, n (%) |  |  | 0.477 |
| Female | 155 (24.1%) | 146 (22.7%) |  |
| Male | 167 (25.9%) | 176 (27.3%) |  |
| Pathologic T stage, n (%) |  |  | 0.272 |
| T1&T2 | 71 (11.1%) | 60 (9.4%) |  |
| T3&T4 | 249 (38.8%) | 261 (40.7%) |  |
| Pathologic N stage, n (%) |  |  | 0.424 |
| N0 | 189 (29.5%) | 179 (28%) |  |
| N1&N2 | 131 (20.5%) | 141 (22%) |  |
| Pathologic M stage, n (%) |  |  | 0.029 |
| M0 | 252 (44.7%) | 223 (39.5%) |  |
| M1 | 36 (6.4%) | 53 (9.4%) |  |

**Table S8.** Relationship between MET expression and clinical pathological characteristics of patients in the TCGA-CRC cohort.

| Characteristics | Low expression of MET (n = 322) | High expression of MET (n = 322) | P-value (χ²) |
| --- | --- | --- | --- |
| Age, n (%) |  |  | 0.524 |
| ≤ 65 | 134 (20.8%) | 142 (22%) |  |
| > 65 | 188 (29.2%) | 180 (28%) |  |
| Gender, n (%) |  |  | 0.133 |
| Female | 160 (24.8%) | 141 (21.9%) |  |
| Male | 162 (25.2%) | 181 (28.1%) |  |
| Pathologic T stage, n (%) |  |  | 0.290 |
| T1&T2 | 71 (11.1%) | 60 (9.4%) |  |
| T3&T4 | 250 (39%) | 260 (40.6%) |  |
| Pathologic N stage, n (%) |  |  | 0.820 |
| N0 | 186 (29.1%) | 182 (28.4%) |  |
| N1&N2 | 135 (21.1%) | 137 (21.4%) |  |
| Pathologic M stage, n (%) |  |  | 0.071 |
| M0 | 247 (43.8%) | 228 (40.4%) |  |
| M1 | 37 (6.6%) | 52 (9.2%) |  |
